# The effects of augmentation choices for locking plate fixation in proximal humerus fracture osteosynthesis: a systematic review and meta-analysis

**DOI:** 10.1186/s10195-025-00852-z

**Published:** 2025-07-17

**Authors:** Hsiao-Yi Cheng, Chun-Wei Liang, Jou-Hua Wang, Yuh-Ruey Kuo, Po-Yen Ko, Chang-Han Chuang, Po-Ting Wu

**Affiliations:** 1 Department of Orthopedics , National Cheng Kung University Hospital, College of Medicine, National Cheng Kung University, Tainan, Taiwan; 2 Department of Primary Care Medicine, Shuang Ho Hospital, Taipei Medical University, Taipei, Taiwan; 3 Department of Psychiatry, Taipei City Psychiatric Center, Taipei City Hospital, Taipei, Taiwan; 4 Department of Orthopaedic Surgery, Show Chwan Memorial Hospital, Changhua, Taiwan; 5 Department of Orthopedics, College of Medicine, National Cheng Kung University, Tainan, Taiwan; 6 Department of Biochemistry and Molecular Biology, College of Medicine, National Cheng Kung University, Tainan, Taiwan; 7 Department of Biomedical Engineering, National Cheng Kung University, Tainan, Taiwan; 8 Medical Device Innovation Center, National Cheng Kung University, Tainan, Taiwan; 9 Orthopedics Department, Tainan Hospital, Ministry of Health and Welfare, Tainan, Taiwan; 10 Department of Orthopedics, National Cheng Kung University Hospital Dou-Liou Branch, Yunlin, Taiwan; 11 Department of Orthopaedic Surgery, Kaohsiung Show Chwan Memorial Hospital, Kaohsiuang, Taiwan

**Keywords:** Proximal humerus fracture, Locking plate, Bone grafts augmentation, Cement augmentation

## Abstract

**Background:**

Various augmentation choices have been reported to improve outcomes following locking plate fixation for proximal humerus fracture, but their effectiveness and safety are still under investigation. This study aims to investigate the effects of augmentation choices, including bone grafts, cement, and intramedullary plates, in locking plate fixation for proximal humerus fractures.

**Methods:**

PubMed, Embase, and Cochrane Library were searched for studies up to April 2024. A random-effects meta-analysis was performed within a frequentist framework.

**Results:**

A total of 35 studies, comprising 6 randomized controlled trials and 29 nonrandomized studies of intervention with a total of 37,494 patients, were included in this review. After adjusting for small-study bias, locking plate fixation with bone grafts or cement did not affect overall complication risk (risk ratio [RR]: 1.03, 95% confidence interval [CI] 0.74–1.45), the screw protrusion risk (RR: 0.74, 95% CI 0.45–1.13), and the avascular necrosis risk (RR: 0.98, 95% CI 0.73–1.32) compared with locking plates alone. Augmentation showed small-to-moderate effects on pain reduction and functional improvement and reduced changes in humeral head height and neck-shaft angle. In subgroup analyses, cement augmentation, while possibly inferior to bone grafts in pain relief and function, showed comparable effects on radiographic outcomes. No significant difference between strut fibular and non-fibular grafts was observed.

**Conclusions:**

Augmentation with bone grafts or cement does not convincingly reduce complication risks or screw protrusion compared with locking plate fixation alone. However, it improves pain, function, and radiographic outcomes in osteosynthesis of proximal humerus fractures.

*Level of evidence*: II.

*Registration:* CRD42024500403.

**Supplementary Information:**

The online version contains supplementary material available at 10.1186/s10195-025-00852-z.

## Introduction

Proximal humerus fractures account for 6% of all fractures and are the third most common fractures in individuals over the age of 65 years [1, 2]. The primary mechanism through which these fractures occur is low-energy trauma, often resulting from a fall at ground level [3]. For older individuals with proximal humerus fracture the mortality rate within 1 year after the injury is 8% [4]. Several treatment options for proximal humerus fracture are available, and the most commonly utilized approaches are nonoperative treatment with sling immobilization, surgical treatment with open reduction and internal fixation (ORIF), intramedullary nailing, and shoulder arthroplasty [5]. The ideal or standard treatment method remains open to debate and depends on patient circumstances and physician judgment [5–8]. 

Several studies have listed relative indications for ORIF involving locking plates [6, 9–12]. However, in older individuals, the complication rates associated with ORIF remain high. The most common complication is intra-articular screw penetration following varus displacement [13]. Intra-articular screw penetration can be caused by poor bone quality and insufficient medial calcar support [14]. Various strategies have been employed to prevent varus collapse, such as the use of inferomedial calcar screws, and augmentation with additional intramedullary plates, bone grafts, or bone cement [15, 16]. Biomechanical models have demonstrated that bone cement enhances the fixation strength of locking plates by augmenting humeral head screws and reducing motion at the bone–implant interface [17–19]. Fibular strut grafts are frequently employed as an endosteal implant to provide additional medial support [20]. Additional types of bone grafts reconstruct the structure of the medial portion of the surgical neck [21]. The objective of these three types of intramedullary augmentation is to strengthen locking plate fixation, reducing the risks of varus deformity and fixation failure. 

To date, there has been no comparison study to evaluate the effects of intramedullary plates. Some systematic reviews [22–24] have investigated the effects of augmentation with fibular strut allografts or bone cement in conjunction with locking plate fixation. Despite promising results, they have limitations owing to small sample sizes and the exclusion of other augmentation techniques. We hypothesized that intramedullary augmentation, specifically using bone grafts, cement, or intramedullary plates in combination with locking plate fixation, would significantly influence the risk of complications, clinical outcomes, and radiographic results in the osteosynthesis of proximal humerus fractures. To test this hypothesis, we conducted an updated meta-analysis encompassing recent clinical studies. 

## Methods

### Search strategy and selection criteria

This systematic review and meta-analysis was planned, conducted, and reported in accordance with the Preferred Reporting Items for Systematic Reviews and Meta-Analyses (PRISMA) guidelines [25]. The protocol of this study was registered on PROSPERO (CRD42024500403).

We searched the PubMed, Embase, and Cochrane Library databases (until April 2024) for randomized controlled trials (RCTs) and nonrandomized studies of intervention (NRSIs) that analyzed the effects of intramedullary augmentation therapy, including bone grafts, bone cement, and intramedullary plates, on locking plate fixation for proximal humerus fractures. Studies with interventions that are not intramedullary augmentations specifically designed for medial support, including dual plates and inferomedial screws, were excluded. The study inclusion criteria—based on the Patient, Intervention, Comparison, Outcomes, and Study (PICOS) criteria—are summarized in Supplementary Method S1. The search strategy is summarized in Supplementary Method S2. 

Two reviewers (H.Y.C. and C.W.L.) independently collected relevant data. Any disagreements regarding trial inclusion and data extraction were resolved through consultation with the other reviewer (P.T.W.). The following data were extracted from each study: bibliography, patient age, patient sex, sample size, fracture type, surgical methods, follow-up duration, and outcomes.

### Risk of bias assessment

Using the revised Cochrane risk-of-bias tool for randomized trials (RoB 2; released on August 22, 2019) and Risk of Bias in Nonrandomized Studies of Interventions (ROBINS-I) tool (released in October 2016), two reviewers (H.Y.C. and C.W.L.) independently assessed the risk of bias of the included studies. Any disagreements were resolved through consultation with the other reviewer (P.T.W.).

### Outcome measures

The primary outcomes were the risks of complications, including the overall risk of complications (the risk of all the complications defined and reported by the included studies), risk of screw protrusion, and risk of avascular necrosis of the humeral head. The secondary outcomes were clinical outcomes, including pain and function, and radiographic outcomes, including changes in the humeral head height and neck-shaft angle. The hierarchy of the scales used to measure pain and functional outcomes is described in Supplementary Method S3.

### Data synthesis and analysis

A random-effects meta-analysis was performed using the meta package of R (version 4.0.4). The effect estimates for pain and function are presented as standardized mean difference (SMD) values, and those for radiographic outcomes are presented as mean difference (MD) values. The overall complication risk, risk of screw penetration, and risk of avascular necrosis of the humeral head are presented as risk ratios (RRs). We calculated SMDs by using Hedges’ *g*: 0.2, 0.5, and 0.8 are typically considered small, medium, and large effect sizes, respectively [26]. The effect estimates were reported with 95% confidence intervals (CIs). The data used for the meta-analysis comprised information on post-intervention scores. For studies involving multiple time points, the data obtained at the last follow-up were preferentially used. When standard differences were not reported, we calculated the values on the basis of standard errors, interquartile ranges, *P* values, or 95% CIs by using the method described by the Cochrane Handbook [27] and Wan et al. [28].

Heterogeneity was assessed using τ^2^ statistics. For continuous variables, τ^2^ values of 0.04, 0.09, and 0.16 indicate low, moderate, and high levels of heterogeneity, respectively; whereas, for dichotomous outcomes, the corresponding τ^2^ values are 0.04, 0.16, and 0.36 [29]. To investigate the causes of heterogeneity, we performed subgroup analyses with subgroups based on augmentation type (fibular bone graft, graft of bone from another location, or cement), and study type (RCT or NRSI).

We constructed funnel plots and performed Egger regression asymmetry tests to detect small-study bias [30]. A *P* value of < 0.1 indicated significant small-study bias. For outcomes exhibiting significant small-study biases, we applied the trim and fill method. This involved excluding studies with small sample sizes (average < 30 patients per treatment arm, regardless of the number of patients excluded from the analysis) and adding hypothetical studies to restore symmetry in the funnel plot [31, 32].

## Results

### Characteristics and risk of bias of the included studies

**Fig. 1:**
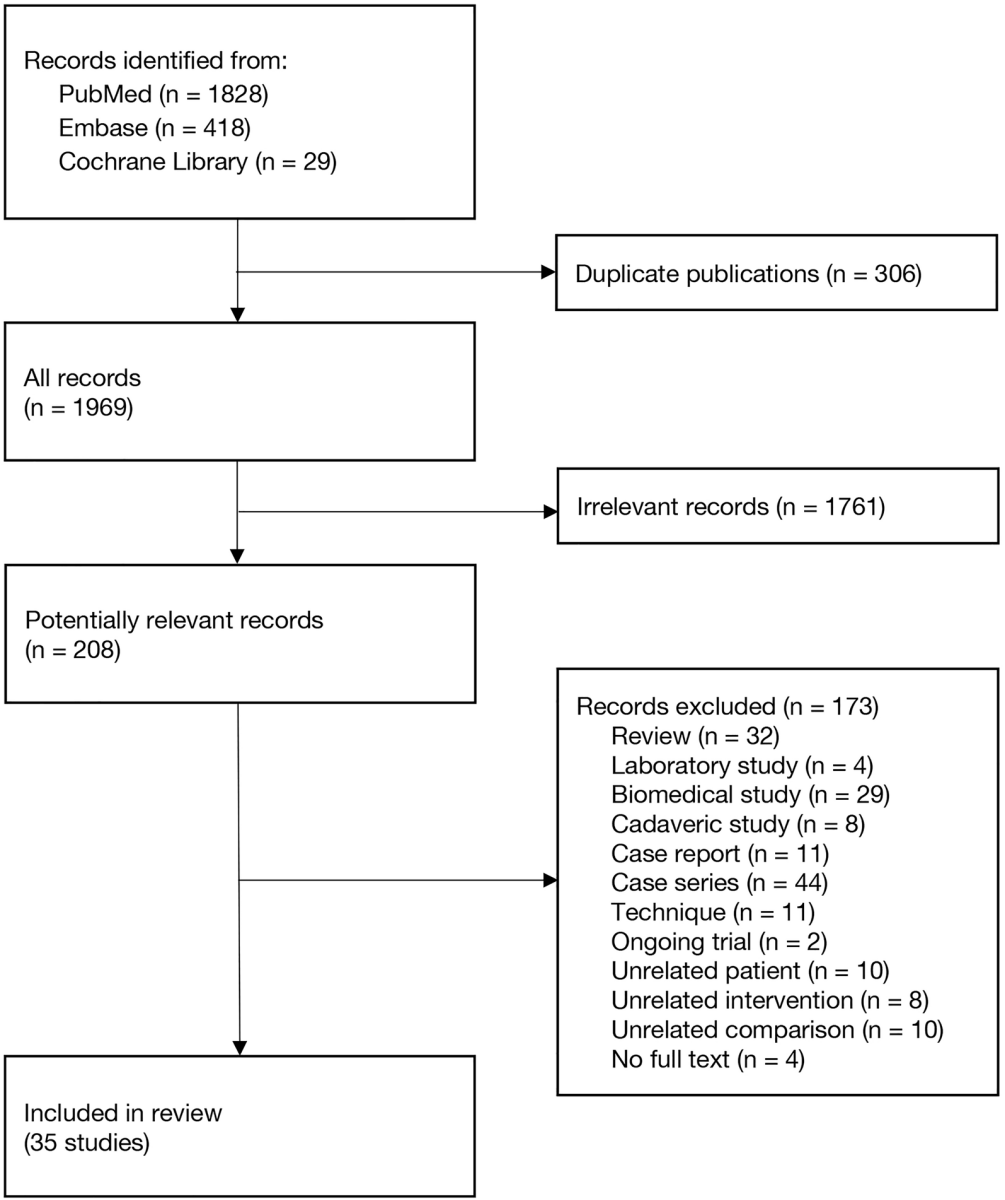
Flow diagram of study inclusion

Figure 1 depicts the study selection process. After eliminating duplicate results and irrelevant articles, we identified 208 articles. Subsequently, 173 articles were excluded (Supplementary Table S1), leaving 35 studies [33–67], comprising 6 RCTs and 29 NRSIs with a total of 37,494 patients (Table 1).

**Table 1:** Characteristics of the included studies

Author	Study design	Groups	*n*	Female ratio	Age (years)	Follow-up duration (months)	Neer classification (2/3/4)
Cha 2017	Retrospective cohort	LP	32	0.75	67.8	15.0	8/21/3
		LP + allograft (fibula, ulna, radius)	20	0.72	71.3	15.0	3/15/2
Chen 2015	Retrospective cohort	LP + allograft (nonspecific)	7	0.57	73.1	30.9	0/0/7
		LP + allograft (fibula)	15	0.60	64.4	34.3	0/0/15
Chen 2018	Retrospective cohort	LP	42	0.64	69.1	35.2	0/10/32
		LP + allograft (fibula)	47	0.74	68.6	33.5	0/12/35
Cui 2019	Retrospective cohort	LP	35	0.69	72.5	32.2	0/25/10
		LP + allograft (fibula)	25	0.72	73.2	31.6	0/17/8
Davids 2020	Retrospective cohort	LP	75	0.70	59.9	17.6	40/35/0
		LP + allograft (fibula)	27	0.70	59.9	17.6	13/14/0
Egol 2012	Retrospective cohort	LP	36	NA	58.0	12.0	16/20/0
		LP + bone cement	27	NA	61.0	12.0	5/18/4
		LP + allograft (cancellous chips)	29	NA	64.0	12.0	3/20/6
Foruria 2021	Retrospective cohort	LP	90	0.79	76.0	43.0	13/48/13
		LP + bone cement	78	0.83	76.0	24.0	12/26/32
Hakimi 2021	Retrospective cohort	LP	53	0.83	74.5	28.5	25/28/0
		LP + bone cement	48	0.83	72.8	27.5	6/42/0
Halvachizadeh 2020	Retrospective cohort	LP	143	0.77	77.7	8.2	111/19/6/3
		LP + allograft (cancellous bone)	24	0.83	77.3	8.4	16/6/2/0
Hengg 2019	RCT	LP	34	0.85	76.1	12.0	5/17/12
		LP + bone cement	33	0.76	77.5	12.0	1/16/15
Karslioglu 2023	RCT	LP + autograft (pectoralis major pedicle bone)	17	0.65	66.3	24.0	0/0/17
		LP + autograft (iliac crest)	17	0.71	67.2	24.0	0/0/17
Katthagen 2018	Retrospective case–control	LP	24	0.92	73.9	12.0	NA
		LP + bone cement	24	0.92	74.2	12.0	NA
Kim 2020	Retrospective cohort	LP	39	0.82	68.1	15.9	20/19/0
		LP + allograft (fibula)	38	0.87	69.8	17.2	24/14/0
Kim 2022	Retrospective cohort	LP	29	0.90	73.8	18.6	14/15/0
		LP + allograft (fibula)	34	0.88	76.0	16.2	15/19/0
Knapp 2023	Retrospective case–control	LP	11	NA	NA	NA	NA
		LP + bone graft (calcium phosphate)	11	NA	NA	NA	NA
Lee 2019	Retrospective cohort	LP	52	0.73	73.3	14.2	25/22/5
		LP + allograft (fibula)	45	0.73	75.6	13.6	21/20/4
Liu 2011	RCT	LP	21	0.80	69.7	18.0	10/8/3
		LP + bone graft (calcium sulfate)	29	0.69	70.4	16.0	9/13/7
Liu 2021	Retrospective cohort	LP	24	0.54	72.2	12.0	0/14/10
		LP + allograft (ilium)	19	0.47	70.3	12.0	0/8/11
		LP + allograft (fibula)	21	0.43	70.8	12.0	0/13/8
Liu 2023	Retrospective cohort	LP	25	0.68	64.8	20.7	0/15/10
		LP + autograft (fibula)	22	0.73	65.8	18.6	0/12/10
Ma 2024	Retrospective cohort	LP	46	0.55	73.6	15.1	16/22/13
		LP + bone cement	51	0.54	71.4	14.5	21/18/7
Myers 2020	Retrospective cohort	LP	72	0.57	54.3	6.5	47/25/0
		LP + allograft (fibula)	61	0.88	62.3	7.8	35/26/0
Opperman 2023	Retrospective case–control	LP	12	0.42	61.0	34.0	0/4/8
		LP + allograft (fibula)	12	0.33	58.0	13.0	0/5/7
Pan 2021	Retrospective cohort	LP	20	0.65	70.2	3.0	0/8/12
		LP + allograft	30	0.60	69.7	3.0	0/13/17
Peng 2012	Retrospective cohort	LP	35	0.77 (all)	74.2 (all)	18.0 (all)	43/46/1 (all)
		LP + allograft (radial shaft, ulnar shaft, humeral shaft, tibial shaft, fibular shaft, and femoral shaft)	55				
Rischen 2023	Retrospective cohort	LP	32,952	0.84	78.0	NA	NA
		LP + autograft	76	0.83	78.0	NA	NA
		LP + allograft	764	0.83	78.0	NA	NA
		LP + bone cement	1,143	0.83	78.0	NA	NA
Sheng 2021	Retrospective cohort	LP + allograft (cancellous bone)	28	0.46	64.0	13.8	0/16/12
		LP + autograft (fibula)	27	0.67	64.0	15.3	0/15/12
Sheng 2023	Retrospective cohort	LP	62	0.57	51.0	25.0	21/25/16
		LP + allograft (cancellous bone)	49	0.66 (all allograft)	52.0 (all allograft)	26.0 (all allograft)	12/32/12
		LP + allograft (fibula)	13				
Siebenbürger 2019	Retrospective cohort	LP	55	0.78	76.6	24.0	20/22/13
		LP + bone cement	39	0.82	78.2	24.0	16/15/8
Tuerxun 2020	Retrospective cohort	LP	22	0.59	64.1	16.3	3/10/19
		LP + allograft (fibula)	41	0.70	64.1	19.3	6/19/16
Wang 2013	RCT	LP	40	0.55	65.4	NA	7/23/10
		LP + bone graft	40	0.50	68.4	NA	5/22/13
Wang 2019	Retrospective cohort	LP	46	0.72	72.5	19.0	0/0/46
		LP + allograft (fibula)	39	0.59	72.2	19.0	0/0/39
Wang 2023	RCT	LP	41	0.68	66.9	24.0	10/17/14
		LP + allograft (fibula)	39	0.61	64.0	24.0	10/14/15
Zhang 2019	RCT	LP	38	0.53	71.4	NA	0/23/15
		LP + allograft (femoral head)	42	0.55	70.9	NA	0/26/16
Zhao 2019	Retrospective cohort	LP	21	0.43	69.0	12.0	0/15/6
		LP + allograft (fibula)	21	0.48	68.8	12.0	0/14/7
Zhu 2014	Cohort	LP	22	0.41	51.5	25.4	0/0/22
		LP + autograft (iliac crest)	18	0.39	51.1	25.4	0/0/18

*LP* locking plate, *RCT* randomized controlled trial

The included studies were published between 2011 and 2024; approximately 75% of the included studies were published between 2019 and 2023. The average follow-up duration was 18.8 months (ranging from 3.0 to 43.0 months). In most studies, the mean age of patients was between 60 and 80 years. Overall, 17 studies involved the use of fibular strut allografts; 17 studies involved other types of bone grafts, including autografts and allografts from other parts of the body; 8 studies involved the use of bone cement; and none of the studies involved the use of intramedullary plates. The results of the risk-of-bias assessment are presented in Supplementary Table S2.

### Small-study bias

**Fig. 2:**
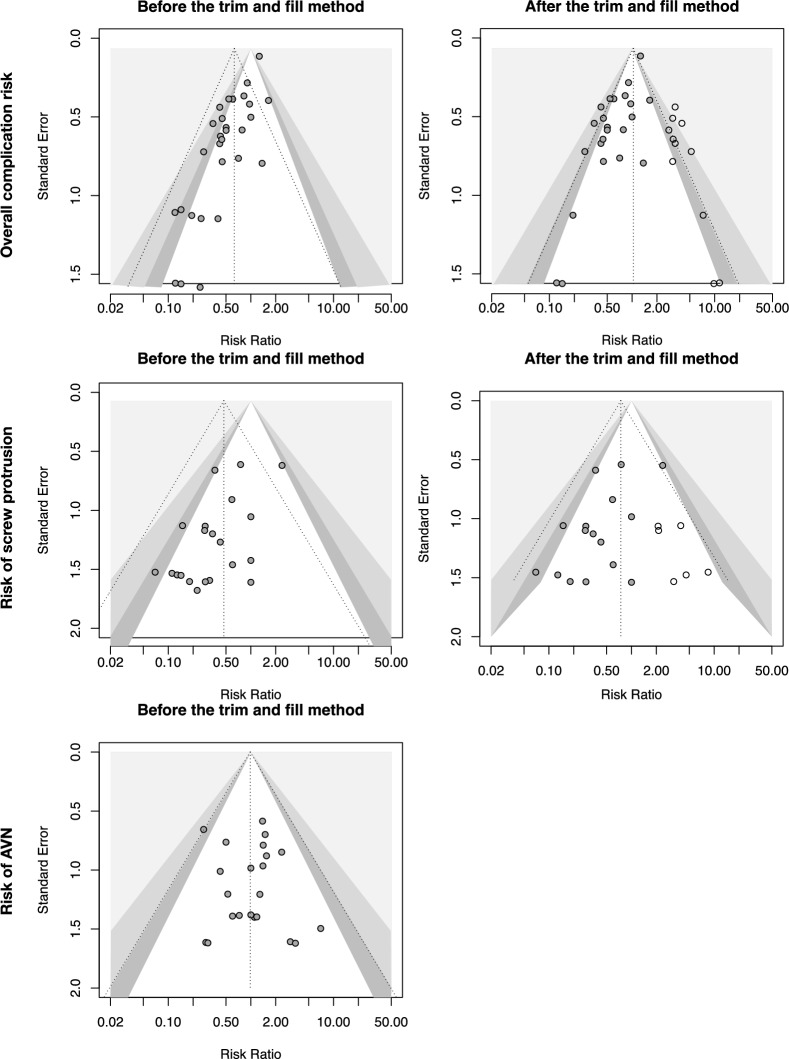
Contour-enhanced funnel plots of the primary outcomes. The contour from dark gray to light gray shows the regions of *P* < 0.1, < 0.05, and < 0.01, respectively. Each black dot represents an effect estimate of a study. The dashed line indicates the effect estimate of the meta-analysis. The white dot represents a hypothetical study added by the trim and fill method. *AVN* avascular necrosis of the humeral head

The funnel plots and Egger regression asymmetry tests showed significant small-study biases in the risk of overall complications, risk of screw protrusion, and in the clinical outcomes (Fig. 2; Supplementary Table S3 and Fig. S2), indicating that the effects may have been overestimated in the meta-analysis of these outcomes. The trim and fill method was therefore adopted to address the small-study biases.

### Primary outcome: complication risk

**Fig. 3:**
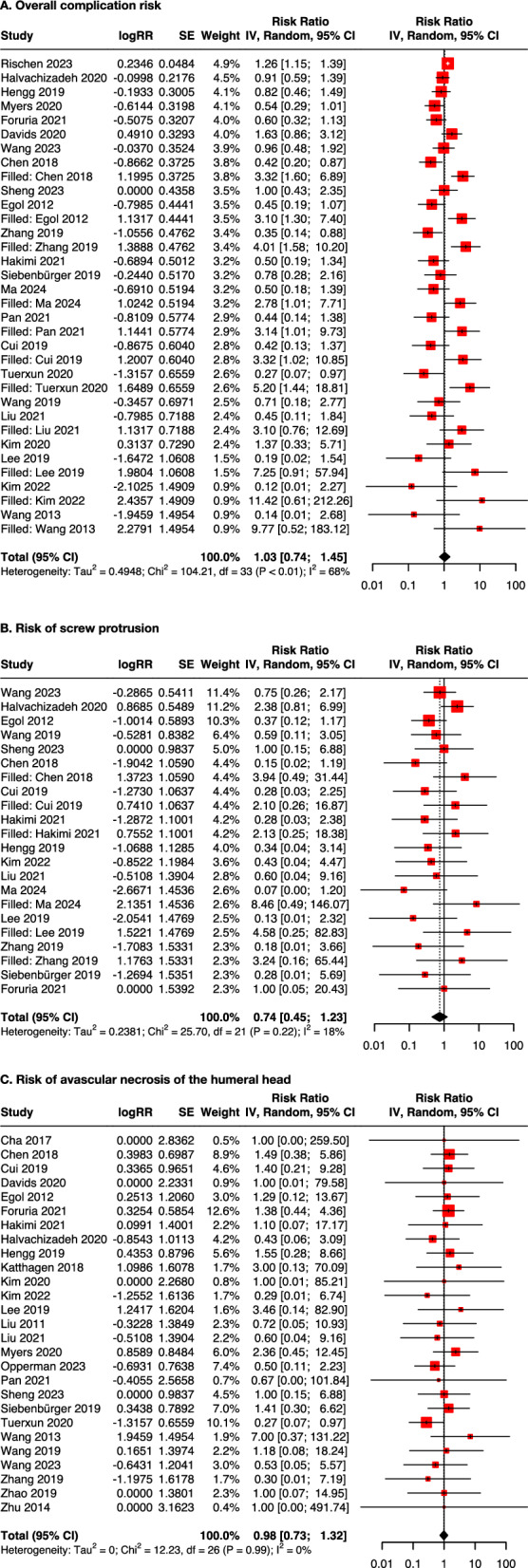
Forest plots adjusted for small-study bias of the primary outcomes. The size of the red box was determined by the weight of the study in the random-effects meta-analysis. *CI* confidence interval, *IV* inverse variance, *RR* relative risk, *SE* standard error

On the basis of 29 studies involving 37,224 patients, the effect estimate using bone grafts or cement in augmentation significantly reduced the overall complication risk compared with using locking plates alone (RR: 0.63, 95% CI 0.50–0.79; Supplementary Table S3 and Fig. S1A). However, after adjusting for small-study bias using the trim and fill method (Fig. 2), this risk reduction was no longer significant (RR: 1.03, 95% CI 0.74–1.45; Table 2 and Fig. 3A). Similar findings were observed for the risk of screw protrusion (RR: 0.74, 95% CI 0.45–1.13; Table 2 and Fig. 3B) and that of avascular necrosis of the humeral head (RR: 0.98, 95% CI 0.73–1.32; Table 2 and Fig. 3C).

**Table 2:** Results adjusted for small-study bias

Outcomes	ES	95% CI	τ^2^
Overall complication risk	RR 1.03	0.74–1.45	0.49
Risk of screw protrusion	RR 0.74	0.45–1.13	0.24
Risk of AVN	RR 0.98	0.73–1.32	0
Pain	SMD −0.34	−0.50 to −0.17	0.09
Function	SMD 0.35	0.15–0.54	0.13
Change of HHH	MD −2.08	−2.78 to −1.37	0.97
Change of NSA	MD −5.39	−7.10 to −3.67	7.08

*AVN* avascular necrosis of the humeral head, *CI* confidence interval, *ES* effect size, *HHH* humeral head height, *NSA* neck-shaft angle, *MD* mean difference, *RR* risk ratio, *SMD* standardized mean difference

A low level of heterogeneity was discovered in the risk of avascular necrosis (τ^2^ = 0), and a moderate level of heterogeneity was found in the overall risk of complications and the risk of screw protrusion (τ^2^ = 0.14 and 0.21, respectively; Supplementary Table S3). Subgroup analyses based on the type of augmentation showed no significant differences in the comparisons between bone grafts and cement or between fibular grafts and non-fibular grafts. In addition, the subgroup analysis by study type revealed no significant differences in complication rates between results from RCTs and those from NRSIs (Supplementary Table S4A–C).

### Secondary outcomes: clinical outcomes

On the basis of 19 studies involving 1758 patients, the effect estimate indicated that augmentation therapy exerted a small-to-moderate effect on pain reduction and functional improvement compared with locking plates alone (SMD: −0.41, 95% CI −0.60 to −0.23; SMD: 0.52, 95% CI 0.33–0.17, respectively; Supplementary Table S3 and Fig. 1D, E). After adjusting for small-study bias using the trim and fill method (Supplementary Fig. S2A, B), the superiority of augmentation therapy over locking plates alone remained evident (pain SMD: −0.34, 95% CI −0.50 to −0.17; function SMD: 0.35, 95% CI: 0.15 to 0.54; Table 2).

A moderate-to-high level of heterogeneity was discovered in both analyses (pain τ^2^ = 0.14; function τ^2^ = 0.16; Supplementary Table S3). Subgroup analyses indicated that bone grafts may lead to greater pain relief and functional improvement (pain *P* = 0.001; function *P* = 0.008) than did cement augmentation. In the comparison of fibular strut grafts and non-fibular grafts, both types resulted in equivalent improvements in pain and function. The subgroup analysis of study type revealed no significant differences between the results from RCTs and those from NRSIs (Supplementary Table S4D, E).

### Secondary outcomes: radiographic outcomes

On the basis of 11 studies involving 819 patients, the effect estimate indicated that augmentation with bone grafts significantly reduced the change in the humeral head height (MD: −2.08, 95% CI −2.78 to −1.37; Supplementary Table S3 and Fig. 1F). In addition, on the basis of 16 trials involving 1209 patients, the effect estimate revealed a significant reduction in the change of the neck-shaft angle (MD: −5.39, 95% CI −7.10 to −3.67; Supplementary Table S3 and Fig. 1G) when compared with the control group. Because no significant small-study bias was detected, the trim and fill method was not further applied (Table 2, Supplementary Fig. S2C, D).

Subgroup analyses comparing bone grafts and cement augmentation, and those comparing fibular strut allografts with non-fibular grafts, showed no significant differences between the options. In addition, when considering study type, there were no significant disparities in changes in humeral head height between RCTs and NRSIs. Notably, while NRSIs exhibited a significant decrease in changes in neck-shaft angle, this finding was not corroborated by the RCT [64] (Supplementary Table S4F, G).

## Discussion

In this systematic review, we identified 35 studies involving 37,494 patients. Although the meta-analysis of all studies initially showed a reduced risk of overall complications and screw protrusion with augmentation using bone grafts and cement compared with locking plate fixation alone, the effects became nonsignificant after adjusting for small-study bias. In terms of clinical outcomes, the augmentation group showed small-to-moderate improvements in pain reduction and functional outcomes compared with the control group. Regarding radiographic outcomes, the meta-analysis indicated that augmentation with bone grafts significantly reduced changes in humeral head height and neck-shaft angle compared with locking plates alone.

Complications following ORIF for proximal humerus fractures have been reported in several studies. The most common complication is intra-articular screw protrusion through the humeral head, which primarily results from the collapse of the humeral head, varus malalignment, and fixation failure [13]. Additional mechanical strength and medial support can be provided to the locking plate fixation when augmented with bone grafts or cement. Our meta-analysis indicated that locking plate fixation augmented with bone grafts or cement results in significantly smaller postoperative changes in the humeral head height and neck-shaft angle, which may further reduce the risk of screw penetration. The radiographic results of our meta-analysis are consistent with those of previous studies [22, 23]. However, owing to small-study bias, the current evidence does not support a significant reduction in the risk of screw penetration as well as that of other complications.

Considering the types of augmentations, although cement augmentation may be less effective than bone grafts for pain relief and functional improvement, it provides comparable benefits in reducing postoperative changes of humeral head height and neck-shaft angle. Hence, cement remains a viable alternative, especially for hospitals without access to a human tissue bank.

Regarding the types of bone grafts, previous systematic reviews focused on the advantages of fibular allografts [22, 23]. Our results show that all bone grafts, including fibular and non-fibular, provide comparable advantages in clinical and radiographic outcomes. Although the use of the strut fibular graft is technique-demanding, especially with the deltoid splitting approach, our findings support the application of bone grafts to enhance medial support, leading to superior outcomes compared with locking plate fixation alone.

In our study, the risk of avascular necrosis, one of the most common complications following proximal humerus fracture fixation, is not significantly increased following augmentation with bone grafts or cement. Consistent with findings in literature, its risk is closely related to the type of fracture pattern [68]. Our results showed that augmentation with bone grafts or cement in plate osteosynthesis of proximal humerus fractures is a safe method with substantial benefits.

Two systematic reviews and meta-analyses have explored the effects of fibular grafts on locking plate fixation for proximal humerus fracture [22, 23]. Neither of these systematic reviews analyzed the outcome of pain reduction. In addition, both reviews employed MDs, instead of SMDs, as the outcome measure for functional outcomes. Because the effect estimates reported by the included studies were based on various scales, the use of MDs substantially limited the amount of data available for analysis. Moreover, both studies focused exclusively on augmentation of fibular allografts and included fewer than ten studies, resulting in low statistical power in the meta-analyses. In the systematic review conducted by Dasari et al. in 2021 [22], a significant improvement in functional outcomes was only observed when using American Shoulder and Elbow Surgeons scores for evaluation. No significant difference in final Constant–Murley scores was found between the augmentation and control groups. However, in the systematic review conducted by Nie et al. in 2022 [23], significant improvements were found in both measure scales. In our meta-analysis, we employed a comprehensive approach by combining different scales of functional outcomes and types of augmentation. We included 21 more studies in our meta-analysis and revealed a significant enhancement in physical function with augmentation compared with locking plate fixation alone.

Our study has some limitations. First, although we addressed small-study bias using statistical methods, these methods could not account for all causes of funnel plot asymmetry, such as genuine heterogeneity or random chance. Therefore, results for these outcomes should be interpreted with caution. Second, the inclusion of mostly NRSIs made the analysis susceptible to confounding bias. Third, high heterogeneity in radiographic outcomes, which could not be fully explained by subgroup analyses, limited the interpretability of those results.

## Conclusions

In proximal humerus fracture osteosynthesis with locking plates, augmentation with bone grafts or cement does not convincingly reduce the overall complication risk or the risk of screw protrusion but provides benefits in pain relief, functional outcomes, and radiographic parameters (reduced changes in humeral head height and neck-shaft angle). Although cement augmentation may be less effective than bone grafts for clinical outcomes, both techniques yield comparable radiographic improvements. No significant difference between strut fibular and non-fibular grafts was observed. In addition, the risk of avascular necrosis does not increase with augmentation. Further randomized controlled trials are needed to validate these findings.

## Supplementary Information

Supplementary material 1.

## Data Availability

All datasets are presented in the main paper and the supplementary files.

## Abbreviations

CI: Confidence interval
MD: Mean difference
NRSI: Nonrandomized studies of intervention
RCT: Randomized controlled trial
RR: Risk ratio
SMD: Standardized mean difference
