# Supplementary material for: The effects of augmentation choices for locking plate fixation in proximal humerus fracture osteosynthesis: a systematic review and meta-analysis
Source: J Orthop Traumatol. 2025 Jul 17;26:47. doi: 10.1186/s10195-025-00852-z (PMC12271005; doi:10.1186/s10195-025-00852-z)
Supplement: Supplementary file 1 — Supplementary material 1. [file 10195_2025_852_MOESM1_ESM.pdf]

Table of contents

Method S1. Eligibility criteria
Method S2. Search strategy
Method S3. Hierarchy for scales
Table S1. List of excluded studies
Table S2. Risk of bias of included studies
Table S3. Results from all the included studies
Table S4. Subgroup analysis of all the included studies
Figure S1. Forest plots from all the included studies
Figure S2. Contour-enhanced funnel plots

Method S1. Eligibility criteria

PICOS

- Patient: radiographically diagnosed of proximal humerus fracture
- Intervention: surgical intervention with locking plate fixation and intramedullary augmentation therapy, including different types of bone grafts, bone cement, and intramedullary plates
- Comparison: surgical intervention with locking plate fixation alone
- Outcome: complication risk (overall complications, humeral head necrosis and screw protrusion), clinical outcomes (pain and function), and radiographic outcomes (change of humeral head height and change of neck-shaft angle)
- Study: randomized controlled trials or observational studies

Method S2. Search strategy

PubMed

(Proximal humeral fracture OR proximal humerus fracture)
AND
(fibular strut graft OR fibular strut OR fibular graft OR fibula OR allograft OR bone graft OR femoral head OR autograft OR medial plate OR cement augmentation OR bone cement OR cementation OR cement OR augment OR augmentation OR reinforcement)

Cochrane Library

#1 Proximal humeral fracture
#2 MeSH descriptor: [Shoulder Fractures] explode all trees
#3 fibular strut graft
#4 fibular strut
#5 fibular graft
#6 MeSH descriptor: [Fibula] explode all trees
#7 MeSH descriptor: [Allografts] explode all trees
#8 MeSH descriptor: [Bone Transplantation] explode all trees
#9 MeSH descriptor: [Femur Head] explode all trees
#10 MeSH descriptor: [Autografts] explode all trees
#11 MeSH descriptor: [Bone Cements] explode all trees
#12 cement augmentation
#13 MeSH descriptor: [Cementation] explode all trees
#14 augment
#15 augmentation
#16 reinforcement
#17 medial plate
#18 #1 OR #2
#19 #3 OR #4 OR #5 OR #6 OR #7 OR #8 OR #9 OR #10 OR #11 OR #12 OR #13 OR #14 OR #15 OR #16 OR #17
#20 #18 AND #19

Embase

('proximal humerus fracture'/exp OR 'humerus proximal fracture' OR 'proximal humeral fracture' OR 'proximal humerus fracture') AND ('fibula graft'/exp OR 'fibula autograft' OR 'fibula flap' OR 'fibula flaps' OR 'fibula graft' OR 'fibula grafts' OR 'fibula osteocutaneous flap' OR 'fibula osteocutaneous flaps' OR 'fibula transplant' OR 'fibula transplantation' OR 'fibular bone flap' OR 'fibular bone flaps' OR 'fibular bone graft' OR 'fibular bone grafts' OR 'fibular flap' OR 'fibular flaps' OR 'fibular graft' OR 'fibular grafts' OR 'fibular osteocutaneous flap' OR 'fibular osteocutaneous flaps' OR 'fibula'/exp OR 'crural bone' OR 'fibula' OR 'fibular body' OR 'os peroneum' OR 'peroneal bone' OR 'allograft'/exp OR 'allo inplant' OR 'allogeneic graft' OR 'allograft' OR 'allografts' OR 'alloplastic graft' OR 'alloplastic implant' OR 'allotransplant' OR 'graft, allogenic' OR 'graft, homologous' OR 'homograft' OR 'homograft sensitivity' OR 'homologous graft' OR 'homotransplant' OR 'transplant, homo' OR 'femoral head'/exp OR 'caput femoris' OR 'femoral head' OR 'femur head' OR 'bone graft'/exp OR 'autograft, bone' OR 'autograft, spongy bone' OR 'autologous bone graft' OR 'bone autograft' OR 'bone flap' OR 'bone flaps' OR 'bone graft' OR 'bone grafts' OR 'bone transplant' OR 'compact bone autograft' OR 'free bone graft' OR 'graft, bone' OR 'osseous flap' OR 'osseous flaps' OR 'osseous graft' OR 'osseous grafts' OR 'osteoarticular graft' OR 'rib autograft' OR 'spongy bone autograft' OR 'viable bone graft' OR 'cement augmentation'/exp OR 'bone cement'/exp OR 'cmw' OR 'cmw cement' OR 'cemex' OR 'cortoss' OR 'jectos' OR 'osteo-firm' OR 'bone cement' OR 'bone cements' OR 'drug free bone cement' OR 'drug free orthopaedic cement' OR 'non medicated orthopedic cement' OR 'non-antimicrobial orthopaedic cement' OR 'non-antimicrobial orthopedic cement' OR 'orthopaedic cement, non-antimicrobial' OR 'orthopaedic cement, non-medicated' OR 'orthopedic cement, non-antimicrobial' OR 'cement'/exp OR 'adhesor cement' OR 'canal cement' OR 'cement' OR 'cement granule' OR 'cementing substance' OR 'cermet cements' OR 'coe tray cement' OR 'portland 500' OR 'augmentation'/exp)

Method S3. Hierarchy for scales

Pain

If a trial presented pain outcomes on more than one scale, we employed the following hierarchical list to extract data from the scale highest on the list:
(1) Global pain, assessed by any scales (visual analog scales, numeric rating scale, or Likert scale)
(2) Pain subscale of Constant-Murley Scores
(3) Algofunctional composite scores as listed below
(4) Patient’s global assessment
(5) Physician's global assessment

Function

Our secondary efficacy outcome was physical function. If a trial presented function outcomes on more than one scale, we used the following hierarchical list to extract data from the scale highest on the list
(1) Constant-Murley Scores (CMS)
(2) American Shoulder and Elbow Surgeons (ASES) score
(3) Shoulder Pain and Disability Index (SPADI)
(4) Disabilities of the arm, shoulder and hand (DASH) score or shortened QuickDASH score
(5) Neer score
(6) EuroQol 5-dimension scale health-related “quality of life” score
(7) Other algofunctional composite scores
(8) Global pain, assessed by any scales (visual analog scales, numeric rating scale, or Likert scale)
(9) Pain subscale of Constant-Murley Scores
(10) Patient’s global assessment
(11) Physician's global assessment

Table S1. List of excluded studies

Reason for exclusion	Title	First Author	Publication Year
Biomechanical study	Proximal humerus fractures: a comparative biomechanical analysis of intra and extramedullary implants	Fechtmeier B	2007
Biomechanical study	Biomechanical analysis of proximal humeral fixation using locking plate fixation with an intramedullary fibular allograft	Mathison C	2010
Biomechanical study	The biomechanical performance of locking plate fixation with intramedullary fibular strut graft augmentation in the treatment of unstable fractures of the proximal humerus	Bae JH	2011
Biomechanical study	The effect of screw augmentation on implant anchorage in proximal humeral head fractures	Schmoelz W	2012
Biomechanical study	The effect of in situ augmentation on implant anchorage in proximal humeral head fractures	Unger S	2012
Biomechanical study	Biomechanical Considerations for Surgical Stabilization of Osteoporotic Fractures	Bogunovic L	2013
Biomechanical study	Investigation of metallic and carbon fibre PEEK fracture fixation devices for three-part proximal humeral fractures	Feerick EM	2013
Biomechanical study	Biomechanical evaluation of locking plate fixation of proximal humeral fractures augmented with calcium phosphate cement	Gradl G	2013
Biomechanical study	Biomechanical comparison of an angular stable plate with augmented and non-augmented screws in a newly developed shoulder test bench	Kathrein S	2013
Biomechanical study	Effect of calcium triphosphate cement on proximal humeral fracture osteosynthesis: a cadaveric biomechanical study	Kennedy J	2013
Biomechanical study	Effect of calcium triphosphate cement on proximal humeral fracture osteosynthesis: a finite element analysis	Kennedy J	2013
Biomechanical study	Biomechanical in vitro assessment of screw augmentation in locked plating of proximal humerus fractures	Raderer G	2013
Biomechanical study	Biomechanical effects of calcar screws and bone block augmentation on medial support in locked plating of proximal humeral fractures	Katthagen JC	2014
Biomechanical study	Biomechanical evaluation of the effect of intramedullary fibular graft in proximal humeral fractures	Bulut T	2017
Biomechanical study	Finite element analysis of an intramedulary anatomical strut for proximal humeral fractures with disrupted medial column instability: A cohort study	Chen H	2020
Biomechanical study	Reconstruction of proximal humeral fractures with a reduced number of screws and a reinforced bone substitute	Cristofolini L	2020
Biomechanical study	Finite element analysis of different fixation methods for poor medial column support proximal humeral fracture	Liu Y	2020
Biomechanical study	Cement augmentation of calcar screws may provide the greatest reduction in predicted screw cut-out risk for proximal humerus plating based on validated parametric computational modelling: Augmenting proximal humerus fracture plating	Varga P	2020
Biomechanical study	Augmented cerclage wire improves the fixation strength of a two-screw construct for humerus split type greater tuberosity fracture: a biomechanical study	Chang CJ	2021
Biomechanical study	Biomechanical study of Proximal humeral fracture fixation: Locking plate with medial support screw vs. locking plate with intramedullary fibular graft	Jang Y	2021
Biomechanical study	[Augmentation in the treatment of proximal humeral and femoral fractures]	van Veelen NM	2022
Biomechanical study	The biomechanical effect of fibular strut grafts on humeral surgical neck fractures with lateral wall comminution	Chang HH	2023
Biomechanical study	[Translated article] Comparative biomechanical study of two configurations of cemented screws in a simulated proximal humerus fracture fixed with locking plate	Martinez-Catala n N	2023
Biomechanical study	Comparative biomechanical study of two configurations of cemented screws in a simulated proximal humerus fracture fixed with locking plate	Martinez-Catala n N	2023
Biomechanical study	Preventing varus collapse in proximal humerus fracture fixation: 90-90 dual plating versus endosteal fibular allograft strut	Patel R	2023
Biomechanical study	Comparison of fibula strut and calcium phosphate cement augmentation of the medial buttress in 2-part proximal humerus fractures reconstruction: a biomechanical study	Pokhvashchev D	2023
Biomechanical study	Finite element analysis of PMMA bone cement reinforced screw plate fixation for osteoporotic proximal humeral fracture	Wang WB	2023
Biomechanical study	[Finite element analysis of PMMA bone cement reinforced screw plate fixation for osteoporotic proximal humeral fracture]	Wang WB	2023
Biomechanical study	Finite element analysis of different fibular support methods to reconstruct the poor medial column of humeral proximal fractures	Yan L.	2023

Cadaveric study	Treatment of Metaphyseal Defects in Plated Proximal Humerus Fractures with a New Augmentation Technique-A Biomechanical Cadaveric Study	Zhelev D	2023
Cadaveric study	Proximal humeral fracture fixation: locking plate construct ∮ intramedullary fibular allograft	Chow RM	2012
Cadaveric study	Intramedullary cortical bone strut improves the cyclic stability of osteoporotic proximal humeral fractures	Hsiao CK	2017
Cadaveric study	Augmentation of a Locking Plate System Using Bioactive Bone Cement-Experiment in a Proximal Humeral Fracture Model	Kuang GM	2018
Cadaveric study	Mechanical Effects of Bone Substitute and Far-Cortical Locking Techniques in 2-Part Proximal Humerus Fracture Reconstruction: A Cadaveric Study	Hast MW	2020
Cadaveric study	Evaluation of Rotator Cuff Augmentation of Proximal Humerus Fracture Fixation	El-Gazzar Y	2022
Cadaveric study	Primary stability of cement augmentation in locking plate fixation for proximal humeral fractures: A comparison of absorbable versus non-absorbable cement	Sch?bel T	2022
Cadaveric study	How to improve the biomechanical stability of endosteal augmentation for proximal humerus fracture with osteopenia? A cadaveric study	Zhu Z	2023
Case report	Immediate reconstruction of bone and skin defects of the humerus with free fibular graft and muscle flap	Sadove RC	1990
Case report	Interprosthetic humeral fracture revision using a tibial allograft total elbow prosthetic composite in a patient with hemophilia A : a case report	Leblanc J	2012
Case report	Fresh Osteochondral Allograft to the Humeral Head for Treatment of an Engaging Reverse Hill-Sachs Lesion: Technical Case Report and Literature Review	Black LO	2016
Case report	Locking plate fixation with femoral head allograft for treatment of nonunion of the surgical neck of the humerus: A case report	Nakamura G	2016
Case report	?alf-folded??pedicled scapular bone flap for nonunion after humeral neck fracture: A case report	Matsumae G	2017
Case report	Fixation of a Proximal Humerus Fracture Using a Polyaxial Locking Plate and Endosteal Fibular Strut	Shah KN	2018
Case report	PHILOS plate fixation with polymethyl methacrylate cement augmentation of an osteoporotic proximal humerus fracture	Kim DY	2020
Case report	A case of simultaneous bilateral reverse shoulder arthroplasty for bilateral comminuted proximal humerus fractures in an elderly patient	Iijima Y	2021
Case report	Non vascularised fibula strut graft augmentation for fixation of proximal Humerus 3-part fracture using philos plate and CC screws in a young male	Supe A	2021
Case report	Treatment of elderly comminuted proximal humeral fracture using endosteal anatomical support system: A case report	Chen H	2023
Case report	Periprosthetic humeral fracture revision using a massive allograft in a patient with rheumatoid arthritis: A case report	Kishimoto K	2023
Case series	[Dislocation-fracture of the shoulder. Apropos of 3 cases treated by open repositioning of the free humeral head and bone graft]	CECCALDI P	1962
Case series	[Four part valgus impacted fractures of the upper extremity of humerus: ilium graft reconstruction. Apropos of 8 cases]	Vandenbussche E	1996
Case series	Intramedullary bone-cement fixation for proximal humeral fracture in elderly patients. A report of 5 cases	Matsuda M	1999
Case series	Reconstruction of posttraumatic bone defects of the humerus with vascularized fibular graft	Adani R	2008
Case series	Treatment of chronic nonunions of the humerus with free vascularized fibula transfer: a report of thirteen cases	Chhabra AB	2009
Case series	[Application of PHILOS plate with injectable artificial bone for the treatment of proximal humeral fractures in elderly patients]	Liu XW	2010
Case series	Pathological fractures of the proximal humerus treated with a proximal humeral locking plate and bone cement	Siegel HJ	2010
Case series	Endosteal strut augment reduces complications associated with proximal humeral locking plates	Neviaser AS	2011
Case series	Rate of avascular necrosis following proximal humerus fractures treated with a lateral locking plate and endosteal implant	Neviaser AS	2011
Case series	Outcomes for four-part proximal humerus fractures treated with a locking compression plate and an autologous iliac bone impaction graft	Kim SH	2012
Case series	Pseudarthrosis of the surgical neck of humerus treated by buttressing with a medial cortico-cancellous graft	Maheshwari J	2012
Case series	Locking plate and fibular allograft augmentation in unstable fractures of proximal humerus	Matassi F	2012

Case series	Proximal humeral fractures: the role of calcium sulphate augmentation and extended deltoid splitting approach in internal fixation using locking plates	Somasundaram K	2013
Case series	Results of surgical management of valgus-impacted proximal humerus fractures with structural allografts	Atalar AC	2014
Case series	Intramedullary allograft fibula as a reduction and fixation tool for treatment of complex proximal humerus fractures with diaphyseal extension	Berkes MB	2014
Case series	[Type C2 proximal humeral fracture fixation using locking-plate with an intramedullary fibular allograft]	Chen X	2014
Case series	The impact of preoperative coronal plane deformity on proximal humerus fixation with endosteal augmentation	Little MT	2014
Case series	Early outcomes of proximal humerus fracture fixation with locking plate and intramedullary fibular strut graft	Tan E	2014
Case series	Allogenic bone grafting for augmentation in two-part proximal humeral fracture fixation in a high-risk patient population	Euler SA	2015
Case series	Factors that influence reduction loss in proximal humerus fracture surgery	Jung SW	2015
Case series	Allograft augmentation in proximal humerus fractures	Euler SA	2016
Case series	Clinical and radiological outcomes of unstable proximal humeral fractures treated with a locking plate and fibular strut allograft	Panchal K	2016
Case series	[-Tricalcium Phosphate in the Surgical Treatment of Proximal Humeral Fractures]	Sarmento M	2016
Case series	Os acromiale open reduction and internal fixation: a review of iliac crest autogenous bone grafting and local bone grafting	Atinga M	2018
Case series	Cement augmentation of humeral head screws reduces early implant-related complications after locked plating of proximal humeral fractures	Katthagen JC	2018
Case series	Minimally Invasive Percutaneous Plate Osteosynthesis via a Deltoidsplitting Approach with Strut Allograft for the Treatment of Displaced 3- or 4-part Proximal Humeral Fractures	Noh YM	2018
Case series	Strut Support with Tricortical Iliac Allografts in Unstable Proximal Humerus Fractures: Surgical Indication and New Definition of Poor Medial Column Support	Lee SJ	2019
Case series	Cambridge experience in spontaneous bone regeneration after traumatic segmental bone defect: A case series and review of literature	Abdulkarim A	2020
Case series	Modified Use of a Fibular Strut in the Reduction and Stabilization of 2-Part Osteoporotic Proximal Humerus Fractures	Avilucea FR	2020
Case series	Does cement augmentation of the screws in angular stable plating for proximal humerus fractures influence the radiological outcome: a retrospective assessment	Knierzinger D	2020
Case series	Treatment of 2-Part Proximal Humeral Fractures in Osteoporotic Patients With Medial Calcar Instability Using a PHILOS Plate Plus an Allogeneic Fibula Inserted Obliquely - A Retrospective Study	Cheng H	2021
Case series	Stable osteosynthesis of cage in cage technique for surgical treatment of proximal humeral fractures	Li J	2021
Case series	Bone Allograft and Locking Plate for Severe Proximal Humeral Fractures: Early and Late Outcomes	Polykandriotis E	2021
Case series	PHILOS Plate Plus Oblique Insertion of Autologous Fibula for 2-Part Proximal Humerus Fractures With Medial Column Disruption: A Retrospective Study	Shu Y	2021
Case series	Endosteal plating in proximal humerus fractures: a novel technique and alternative to fibular strut allograft for medial column support	Braman JP	2022
Case series	Clinical and functional outcomes of displaced proximal humerus fractures treated with PHILOS plate system and autologous bone graft	Shah IA	2022
Case series	Fibula Strut Autograft With Locking Plate Construct In Comminuted Proximal Humerus Fractures	Yadav AK	2022
Case series	Fibula flap in upper extremity segmental/critical size bone defects fixed with locking plates. Single-institution observational cohort	Boretto JG	2023
Case series	Efficacy of fibular allograft and locking plate in the treatment of proximal humeral fracture	Dezhi L	2023
Case series	[Treatment of four-part proximal humerus fractures with depressed humeral head collapse using strut allograft with locking plates]	Liu L	2023
Case series	Fibular Autograft as Medial Support with Proximal Humerus Locking Plate Construct in Comminuted Proximal Humerus Fractures: A Retrospective Analysis	Panchal S	2023
Case series	Cement-Augmented Screw Fixation with PHILOS Plating for Osteoporotic Proximal Humeral Fractures: An Observation of Mid- and Long-Term Curative Efficacy	She R	2023

Case series	Application of Intramedullary Calcar Support Plate and Lateral Locking Plate in Elderly Patients with Neer 3 and 4-Part Fractures of Proximal Humerus Through a Deltoid Splitting Approach	Wang Y	2024
Case series	[Clinical effect of allogeneic peroneal bone marrow support combined with plate fixation for the treatment of Neer type Ⅳ proximal humeral fractures]	Sun ZG	2024
Laboratory study	Medial support by fibula bone graft in angular stable plate fixation of proximal humeral fractures: an in vitro study with synthetic bone	Osterhoff G	2011
Laboratory study	In vitro temperature evaluation during cement augmentation of proximal humerus plate screw tips	Blazejak M	2013
Laboratory study	Biomechanical in vitro assessment of screw augmentation in locked plating of proximal humerus fractures	R?derer G	2013
Laboratory study	Augmentation technique on the proximal humerus		2015
No full text	Distal ulna allograft for proximal humerus fractures	Ramirez MA	2016
No full text	Cement in Proximal Humerus Fracture	Molinedo M	2021
No full text	Proximal humeral internal locking system and injectable calcium sulfate graft for the treatment of osteoporotic proximal humeral fractures in elderly patients		2012
No full text	Functional outcomes in proximal humerus fractures: A prospective registry-based analysis	Ahmad T	2021
Ongoing trial	PHILOS Augmented - a Multicenter Randomized Controlled Trial		2013
Ongoing trial	Randomized clinical trial of proximal humeral fractures in three or four parts in elderly: blocked plate with or without synthetic graft augmentation		2021
Review	Decision making in difficult proximal humerus fractures: When to fix, pin, or replace	Braman JP	2004
Review	Evaluation of the osteoporotic proximal humeral fracture and strategies for structural augmentation during surgical treatment	Namdari S	2012
Review	Proximal humeral osteoarticular allografts: technique, pearls and pitfalls, outcomes	Farfalli GL	2015
Review	Augmentation in proximal humeral fractures: When and how?	Lawrence C	2015
Review	Proximal humerus allograft prosthetic composites: technique, outcomes, and pearls and pitfalls	Lozano-Caldera n SA	2015
Review	The use of augmentation techniques in osteoporotic fracture fixation	Kammerlander C	2016
Review	The Applications of Finite Element Analysis in Proximal Humeral Fractures	Ye Y	2017
Review	Locking-plate fixation of proximal humerus fractures in patients over 60 continues to be associated with a high complication rate	Barlow JD	2019
Review	Osteoporotic Bone: When and How to Use Augmentation?	Schuetze K	2019
Review	Indications and results of osteosynthesis for proximal Humerus fragility fractures in elderly patients	Murena L	2020
Review	Effect of anatomic locking plate combined with allograft fibula on proximal humerus fracture: A meta-analysis	Xiang FF	2020
Review	[Research progress in treatment of proximal humeral fracture with fibular allograft and locking plate]	Xing F	2020
Review	Research progress in treatment of proximal humeral fracture with fibular allograft and locking plate	Xing F	2020
Review	Locking plate in the treatment of proximal humeral fractures involving humeral calcar: Effective support, complications and functional recovery	Xu P	2020
Review	[Research progress on medial support augmentation of plate osteosynthesis for proximal humeral fractures]	Chang Z	2021
Review	Research progress on medial support augmentation of plate osteosynthesis for proximal humeral fractures	Chang Z	2021
Review	Molecular enhancement of fracture healing - Is there a role for Bone Morphogenetic Protein-2, parathyroid hormone, statins, or sclerostin-antibodies?	Henssler L	2021
Review	Minimally invasive plate osteosynthesis: An update of practise	van de Wall BJM	2021
Review	Latest Trends in the Current Treatment of Proximal Humeral Fractures - an Analysis of 1162 Cases at a Level-1 Trauma Centre with a Special Focus on Shoulder Surgery	Dey Hazra RO	2022
Review	Interventions for treating proximal humeral fractures in adults	Helen HG Handoll	2022
Review	Augmentation in the treatment of proximal humeral and femoral fractures	van Veelen N.M	2022
Review	[Progress in the research of medial column reconstruction of proximal humerus fractures in the elderly]	Yao CJ	2022

Review	Plate Fixation of Proximal Humerus Fractures: How to Get It Right and Future Directions for Improvement	Foruria AM	2023
Review	Open reduction internal fixation of proximal humerus fractures	Berkes MB	2013
Review	Meta-analysis of locking plate combined with fibular allograft and locking plate alone in the treatment of proximal humeral fractures	Tu D	2020
Review	Fibular Strut Graft Augmentation for Open Reduction and Internal Fixation of Proximal Humerus Fractures: A Systematic Review and the Authors' Preferred Surgical Technique	Saltzman BM	2016
Review	Augmentation of plate osteosynthesis for proximal humeral fractures: a systematic review of current biomechanical and clinical studies	Biermann N	2019
Review	Synthetic Bone Substitutes and Mechanical Devices for the Augmentation of Osteoporotic Proximal Humeral Fractures: A Systematic Review of Clinical Studies	Marongiu G	2020
Review	Improved outcomes for proximal humerus fracture open reduction internal fixation augmented with a fibular allograft in elderly patients: a systematic review and meta-analysis	Dasari SP	2022
Review	Effects of fibular strut augmentation for the open reduction and internal fixation of proximal humeral fractures: a systematic review and meta-analysis	Nie W	2022
Review	Role of Fibular Allograft in Proximal Humerus Fractures: A Systematic Review	Segarra B	2022
Review	[Locking plate fixation with fibular strut allograft versus locking plate fixation alone for the treatment of proximal humeral fractures in adults:a Meta-analysis]	Tang D	2022
Technique	Fixed-angle locked plating of two-, three-, and four-part proximal humerus fractures	Badman BL	2008
Technique	Indirect medial reduction and strut support of proximal humerus fractures using an endosteal implant	Gardner MJ	2008
Technique	Allograft-prosthesis composite reconstruction of the proximal part of the humerus: surgical technique	Abdeen A	2010
Technique	Fixation strategies to prevent screw cut-out and malreduction in proximal humeral fracture fixation	Namdari S	2012
Technique	Reconstruction of Proximal Humeral Defects with Shoulder Arthrodesis Using Free Vascularized Fibular Graft: Surgical Technique	Armangil M	2013
Technique	Calcium phosphate cement augmentation of proximal humerus fractures	Cantlon MB	2013
Technique	Avoiding cement bone necrosis effect on tuberosity healing: The "black-and-tan" technique	Levy JC	2013
Technique	[Augmentation technique on the proximal humerus]	Scola A	2015
Technique	Internal Fixation of 4-Part Proximal Humerus Fractures and Fracture Dislocations Using Extended Deltoid-splitting Approach, Locking Plates, and Calcium Sulfate Graft: Technical Details	Zadeh HG	2018
Technique	Distal Clavicle Autograft in Fractures of the Proximal Humerus: Surgical Technique	De Mello Ribeiro Pinto G	2022
Technique	Technique of Open Reduction and Internal Fixation of Comminuted Proximal Humerus Fractures With Allograft Femoral Head Metaphyseal Reconstruction	Parada SA	2015
Unrelated comparison	[Treatment of Neer type 4 impacted valgus fractures of the proximal humerus with open reduction, elevation, and grafting]	Atalar AC	2007
Unrelated comparison	Geriatric proximal humeral fracture patients show similar clinical outcomes to non-geriatric patients after osteosynthesis with endosteal fibular strut allograft augmentation	Hinds RM	2015
Unrelated comparison	Operative treatment of 2-part surgical neck fractures of the proximal humerus (AO 11-A3) in the elderly: Cement augmented locking plate Philos? vs. proximal humerus nail MultiLoc?	Helfen T	2016
Unrelated comparison	Allogeneic bone grafting strengthens the internal fixation of proximal humeral fractures in high-risk groups	Su ZH	2016
Unrelated comparison	Locking system strengthened by biomimetic mineralized collagen putty for the treatment of osteoporotic proximal humeral fractures	Peng C	2017
Unrelated comparison	A novel cement-reinforced screw combined with locking plate fixation versus humeral head arthroplasty in the treatment of osteoporotic fractures of the proximal humerus	She R	2020
Unrelated comparison	Subgroups and differences of fixation in 3-part proximal humerus fractures	Bekmezci T	2023
Unrelated comparison	Comparison of intramedullary fibular allograft with locking compression plate versus shoulder hemi-arthroplasty for repair of osteoporotic four-part proximal humerus fracture: Consecutive, prospective, controlled, and comparative study	Chen H	2016
Unrelated comparison	Which additional augmented fixation procedure decreases surgical failure after proximal humeral fracture with medial comminution: fibular allograft or inferomedial screws?	Kim DS	2018

Unrelated comparison	Operative treatment of 2-part surgical neck type fractures of the proximal humerus in the elderly: Cement augmented locking plate PHILOS? vs. proximal humerus nail multiloc?	Helfen T	2020
Unrelated intervention	Effectiveness of Adipose Tissue Derived Mesenchymal Stem Cells as Osteogenic Component in Composite Grafts		2012
Unrelated intervention	Does medial support decrease major complications of unstable proximal humerus fractures treated with locking plate?	Jung WB	2013
Unrelated intervention	BMC2012, Cell based therapy by implanted bone marrow-derived mononuclear cells (BMC) for bone augmentation of plate-stabilized proximal humeral fractures - a randomized, open, multicentric study - phase IIa		2015
Unrelated intervention	Safety and feasibility of cell-based therapy of autologous bone marrow-derived mononuclear cells in plate-stabilized proximal humeral fractures in humans	Seebach C	2016
Unrelated intervention	Technical Tips for Reduction and Stable Fixation of Proximal Humerus Fractures	Brodke DJ	2023
Unrelated intervention	Surgical treatment of three and four-part proximal humeral fractures	Solberg BD	2009
Unrelated intervention	Treatment progress on proximal humerus fracture with proximal humerus cage	Lu S	2020
Unrelated intervention	[Effectiveness analysis of proximal humerus internal locking system plate combined with rotator cuff reinforcement suture in treatment of Neer type 12. proximal humerus fracture]	Ma J	2022
Unrelated patient	Vascularized Ulnar Periosteal Pedicled Flap for Upper Extremity Reconstruction in Adults: A Prospective Case Series of 11?Patients	Barrera-Ochoa S	2022
Unrelated patient	Comparison of Nanocrystalline Hydroxyapatite Bone Graft with Empty Defects in Long Bone Fractures: A Retrospective Case-Control Study	Pawelke J	2023
Unrelated patient	Treatment of benign lesions of humerus with resection and non-vascularised, autologous fibular graft	Grzegorzewski A	2010
Unrelated patient	Vascularized fibula grafts for reconstruction of bone defects after resection of bone sarcomas	Petersen MM	2010
Unrelated patient	Implementation of locking compression plate together with intramedullary fibular graft in atrophic type humeral nonunions	Erden ER	2012
Unrelated patient	Strut graft vs. traditional plating in the management of periprosthetic humeral fractures: a multicentric cohort study	Rollo G	2020
Unrelated patient	Outcomes following Free Fibula Physeal Transfer for Pediatric Proximal Humerus Reconstruction: An International Multi-Institutional Study	Azoury SC	2023
Unrelated patient	Are Vascularized Fibula Autografts a Long-lasting Reconstruction After Intercalary Resection of the Humerus for Primary Bone Tumors?	Campanacci DA	2023
Unrelated patient	Outcomes of proximal humeral reconstruction with cemented osteoarticular allograft in pediatric patients: a retrospective cohort study	Jamshidi K	2023
Unrelated patient	Bone cemented K-wire fixation versus elastic stable intramedullary nailing fixation of paediatric proximal humerus fractures: A prospective cohort study	Liu S	2023

Table S2. Risk of bias of included studies

A. Randomized controlled trials: risk of bias evaluated by Cochrane risk-of-bias tool for randomized trials (RoB 2.0)

**Study**	**Bias arising from the randomization process**	**Bias due to deviations from intended interventions**	**Bias due to missing outcome data**	**Bias in measurement of the outcome**	**Bias in selection of the reported result**
Hengg 2019	Low risk	Low risk	Low risk	High risk	Low risk
Karslioglu 2023	Low risk	Low risk	Low risk	Some concerns	Some concerns
Liu 2011	Some concerns	Low risk	Some concerns	High risk	Some concerns
Wang 2013	Some concerns	Low risk	Some concerns	High risk	Some concerns
Wang 2023	Low risk	Low risk	Low risk	High risk	Low risk
Zhang 2019	Some concerns	Low risk	Some concerns	High risk	Some concerns

B. Non-randomized studies of interventions: risk of bias evaluated by Risk of Bias in Non-randomized Studies of Interventions (ROBINS-I)

**Study**	**Bias due to confounding**	**Bias in selection of participants into the study**	**Bias in classification of interventions**	**Bias due to deviations from intended interventions**	**Bias due to missing data**	**Bias in measurment of outcomes**	**Bias in selection of the reported result**
Cha 2017	Moderate risk	Low risk	Low risk	Low risk	Moderate risk	Serious risk	Moderate risk
Chen 2015	Moderate risk	Low risk	Low risk	Low risk	Low risk	Moderate risk	Moderate risk
Chen 2018	Moderate risk	Low risk	Low risk	Low risk	Low risk	Serious risk	Moderate risk
Cui 2019	Moderate risk	Low risk	Low risk	Low risk	Moderate risk	Serious risk	Moderate risk
Davids 2020	Serious risk	Low risk	Low risk	Low risk	Low risk	Serious risk	Moderate risk
Egol 2012	Serious risk	Low risk	Low risk	Low risk	Moderate risk	Serious risk	Moderate risk
Foruria 2021	Moderate risk	Low risk	Low risk	Low risk	Low risk	Serious risk	Moderate risk
Hakimi 2021	Moderate risk	Low risk	Low risk	Low risk	Low risk	Serious risk	Moderate risk
Halvachizadeh 2020	Moderate risk	Low risk	Low risk	Low risk	Low risk	Serious risk	Moderate risk
Katthagen 2018	Moderate risk	Low risk	Low risk	Low risk	Low risk	Serious risk	Moderate risk
Kim 2020	Moderate risk	Low risk	Low risk	Low risk	Moderate risk	Serious risk	Moderate risk
Kim 2022	Moderate risk	Low risk	Low risk	Low risk	Low risk	Serious risk	Moderate risk
Knapp 2023	Moderate risk	Low risk	Low risk	Low risk	Low risk	Serious risk	Moderate risk
Lee 2019	Moderate risk	Low risk	Low risk	Low risk	Low risk	Serious risk	Moderate risk
Liu 2021	Moderate risk	Low risk	Low risk	Low risk	Moderate risk	Serious risk	Moderate risk
Liu 2023	Moderate risk	Low risk	Low risk	Low risk	Moderate risk	Serious risk	Moderate risk
Ma 2024	Moderate risk	Low risk	Low risk	Low risk	Moderate risk	Serious risk	Moderate risk
Myers 2020	Serious risk	Low risk	Low risk	Low risk	Low risk	Moderate risk	Moderate risk
Opperman 2023	Moderate risk	Low risk	Low risk	Low risk	Low risk	Serious risk	Moderate risk
Pan 2021	Moderate risk	Low risk	Low risk	Low risk	Moderate risk	Serious risk	Moderate risk

Peng 2012	Serious risk	Low risk	Low risk	Low risk	Moderate risk	Serious risk	Moderate risk
Rischen 2023	Serious risk	Low risk	Low risk	Low risk	Low risk	Serious risk	Moderate risk
Sheng 2021	Moderate risk	Low risk	Low risk	Low risk	Moderate risk	Moderate risk	Moderate risk
Sheng 2023	Moderate risk	Low risk	Low risk	Low risk	Low risk	Serious risk	Moderate risk
Siebenbürger 2019	Moderate risk	Low risk	Low risk	Low risk	Moderate risk	Serious risk	Moderate risk
Tuerxun 2020	Moderate risk	Low risk	Low risk	Low risk	Low risk	Serious risk	Moderate risk
Wang 2019	Serious risk	Low risk	Low risk	Low risk	Moderate risk	Serious risk	Moderate risk
Zhao 2019	Moderate risk	Low risk	Low risk	Low risk	Low risk	Serious risk	Moderate risk
Zhu 2014	Moderate risk	Low risk	Low risk	Low risk	Moderate risk	Serious risk	Moderate risk

Table S3. Results from all the included studies

Results from all studies						
**Outcomes**	**k**	**n**	**ES**	**95%CI**	**τ^2**	**Egger's test**
Overall complication risk	29	37224	RR 0.63	0.50 to 0.79	0.14	p <0.0001
Risk of screw protrusion	22	1784	RR 0.47	0.31 to 0.71	0.21	p = 0.01
Risk of AVN	27	2192	RR 0.98	0.73 to 1.32	0	p = 0.90
Pain	26	2059	SMD -0.41	-0.60 to -0.23	0.14	p = 0.06
Function	26	2059	SMD 0.52	0.33 to 0.71	0.16	p = 0.03
Change of HHH	11	819	MD -2.08	-2.78 to -1.37	0.97	p = 0.74
Change of NSA	16	1209	MD -5.39	-7.10 to -3.67	7.08	p = 0.11

Abbreviation: CI, confidence interval; ES, effect size; HHH, humeral head height; k, study number; n, patient number; NSA, neck-shaft angle; RCT, randomized controlled trial; RR, risk ratio; SMD, standardized mean difference.

Table S4. Subgroup analysis of all the included studies

A. Overall complication risk

Subgroup	k	n	RR (95%CI)	τ^2	Subgroup difference
**Augmentation type**					
Bone graft	21	1622	0.58 (0.43 to 0.78)	0.15	p = 0.77
Cement	7	667	0.62 (0.45 to 0.85)	0	
**Bone graft origin**					
Fibula	14	1012	0.58 (0.40 to 0.86)	0.18	p = 0.82
Others	8	634	0.54 (0.32 to 0.92)	0.18	
**Study design**					
NRSI	24	36867	0.64 (0.49 to 0.82)	0.15	p = 0.73
RCT	5	357	0.57 (0.30 to 1.06)	0.21	

B. Risk of screw protrusion

Subgroup	k	n	RR (95%CI)	τ^2	Subgroup difference
**Augmentation type**					
Bone graft	15	1117	0.58 (0.32 to 1.05)	0.25	p = 0.19
Cement	7	667	0.30 (0.14 to 0.65)	0	
**Bone graft origin**					
Fibula	10	637	0.47 (0.25 to 0.88)	0	p = 0.27
Others	6	504	0.95 (0.32 to 2.80)	0.40	
**Study design**					
NRSI	19	1557	0.44 (0.28 to 0.71)	0	p = 0.74
RCT	3	227	0.58 (0.23 to 1.44)	0	

C. Risk of avascular necrosis of the humeral head

Subgroup	k	n	RR (95%CI)	τ^2	Subgroup difference
All studies	27	2192	0.98 (0.73 to 1.32)	0	NA
**Augmentation type**					
Bone graft	21	1622	0.82 (0.50 to 1.34)	0	p = 0.20
Cement	6	570	1.44 (0.70 to 2.97)	0	
**Bone graft origin**					
Fibula	14	1012	0.84 (0.47 to 1.52)	0.06	p = 0.89
Others	8	610	0.78 (0.29 to 2.08)	0	
**Study design**					
NRSI	22	1835	0.96 (0.62 to 1.49)	0	p = 0.80
RCT	5	357	1.12 (0.38 to 3.26)	0	

D. Pain

Subgroup	k	n	SMD (95%CI)	τ^2	Subgroup difference
**Augmentation type**					
Bone graft	20	1484	-0.54 (-0.74 to -0.33)	0.14	p = 0.001
Cement	6	575	-0.07 (-0.27 to 0.11)	0.01	
**Bone graft origin**					
Fibula	14	949	-0.45 (-0.65 to -0.25)	0.07	p = 0.41
Others	8	621	-0.64 (-1.06 to -0.22)	0.30	

Study design					
NRSI	21	1702	-0.40 (-0.59 to -0.20)	0.15	p = 0.71
RCT	5	357	-0.49 (-0.92 to -0.06)	0.18	

E. Function

Subgroup	k	n	SMD (95%CI)	τ^2	Subgroup difference
**Augmentation type**					
Bone graft	20	1484	0.63 (0.43 to 0.84)	0.15	p = 0.008
Cement	6	575	0.19 (-0.07 to 0.45)	0.06	
**Bone graft origin**					
Fibula	14	949	0.53 (0.31 to 0.75)	0.10	p = 0.11
Others	8	621	0.87 (0.58 to 1.22)	0.18	
**Study design**					
NRSI	21	1702	0.55 (0.34 to 0.75)	0.16	p = 0.60
RCT	5	357	0.42 (-0.01 to 0.84)	0.18	

F. Change of humeral head height

Subgroup	k	n	MD (95%CI)	τ^2	Subgroup difference
**Augmentation type**					
Bone graft	10	722	-2.15 (-2.84 to -1.45)	1.05	p = 0.08
Cement	1	97	-1.50 (-1.69 to -1.31)	NA	
**Bone graft origin**					
Fibula	8	514	-2.39 (-2.48 to -2.30)	<0.0001	p = 0.52
Others	4	294	-1.89 (-3.41 to -0.38)	2.27	
**Study design**					
NRSI	10	739	-2.16 (-2.82 to -1.51)	0.97	p = 0.14
RCT	1	80	-0.70 (-2.52 to 1.12)	NA	

G. Change of neck-shaft angle

Subgroup	k	n	MD (95%CI)	τ^2	Subgroup difference
**Augmentation type**					
Bone graft	15	1112	-5.35 (-6.97 to -3.73)	8.04	p = 0.54
Cement	1	97	-5.90 (-6.56 to -5.24)	NA	
**Bone graft origin**					
Fibula	13	923	-4.88 (-6.39 to -3.37)	5.46	p = 0.66
Others	4	294	-5.86 (-10.02 to -1.70)	16.54	
**Study design**					
NRSI	15	1129	-5.68 (-7.06 to -4.31)	5.70	p = 0.003
RCT	1	80	2.30 (-2.86 to 7.46)	NA	

Abbreviations: CI, confidence interval; k, study number; n, patient number; NRSI, non-randomized study of intervention; RCT, randomized controlled trial; RR, risk ratio.

Figure S1. Forest plots from all the included studies

A. Overall complication risk

Study	logRR	SE	Weight	Risk Ratio IV, Random, 95% CI
Cha 2017	-0.9163	1.0811	1.1%	0.40 [0.05; 3.33]
Chen 2018	-0.8662	0.3725	5.1%	0.42 [0.20; 0.87]
Cui 2019	-0.8675	0.6040	2.8%	0.42 [0.13; 1.37]
Davids 2020	0.4910	0.3293	5.7%	1.63 [0.86; 3.12]
Egol 2012	-0.7985	0.4441	4.2%	0.45 [0.19; 1.07]
Foruria 2021	-0.5075	0.3207	5.8%	0.60 [0.32; 1.13]
Hakimi 2021	-0.6894	0.5012	3.6%	0.50 [0.19; 1.34]
Halvachizadeh 2020	-0.0998	0.2176	7.6%	0.91 [0.59; 1.39]
Hengg 2019	-0.1933	0.3005	6.2%	0.82 [0.46; 1.49]
Katthagen 2018	-1.3863	1.0801	1.1%	0.25 [0.03; 2.08]
Kim 2020	0.3137	0.7290	2.1%	1.37 [0.33; 5.71]
Kim 2022	-2.1025	1.4909	0.6%	0.12 [0.01; 2.27]
Lee 2019	-1.6472	1.0608	1.1%	0.19 [0.02; 1.54]
Liu 2011	-2.1145	1.0414	1.2%	0.12 [0.02; 0.93]
Liu 2021	-0.7985	0.7188	2.2%	0.45 [0.11; 1.84]
Ma 2024	-0.6910	0.5194	3.5%	0.50 [0.18; 1.39]
Myers 2020	-0.6144	0.3198	5.9%	0.54 [0.29; 1.01]
Opperman 2023	-0.8473	0.5563	3.2%	0.43 [0.14; 1.28]
Pan 2021	-0.8109	0.5774	3.0%	0.44 [0.14; 1.38]
Rischen 2023	0.2346	0.0484	10.0%	1.26 [1.15; 1.39]
Sheng 2023	0.0000	0.4358	4.3%	1.00 [0.43; 2.35]
Siebenbürger 2019	-0.2440	0.5170	3.5%	0.78 [0.28; 2.16]
Tuerxun 2020	-1.3157	0.6559	2.5%	0.27 [0.07; 0.97]
Wang 2013	-1.9459	1.4954	0.6%	0.14 [0.01; 2.68]
Wang 2019	-0.3457	0.6971	2.3%	0.71 [0.18; 2.77]
Wang 2023	-0.0370	0.3524	5.4%	0.96 [0.48; 1.92]
Zhang 2019	-1.0556	0.4762	3.9%	0.35 [0.14; 0.88]
Zhao 2019	-1.9459	1.0235	1.2%	0.14 [0.02; 1.06]
Zhu 2014	-1.4137	1.5171	0.6%	0.24 [0.01; 4.76]
**Total (95% CI)**			**100.0%**	**0.63 [0.50; 0.79]**

Heterogeneity: $Tau^2 = 0.1405$; $Chi^2 = 75.26$, df = 28 ($P < 0.01$); $I^2 = 63\%$

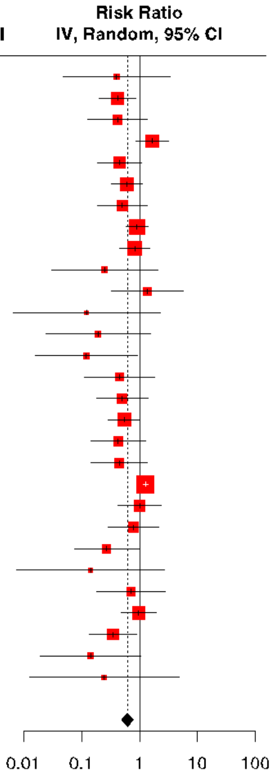

B. Risk of screw protrusion

Study	logRR	SE	Weight	Risk Ratio IV, Random, 95% CI
Cha 2017	-1.1486	1.5233	2.5%	0.32 [0.02; 6.28]
Chen 2018	-1.9042	1.0590	4.9%	0.15 [0.02; 1.19]
Cui 2019	-1.2730	1.0637	4.8%	0.28 [0.03; 2.25]
Egol 2012	-1.0014	0.5893	11.7%	0.37 [0.12; 1.17]
Foruria 2021	0.0000	1.5392	2.5%	1.00 [0.05; 20.43]
Hakimi 2021	-1.2872	1.1001	4.5%	0.28 [0.03; 2.38]
Halvachizadeh 2020	0.8685	0.5489	12.8%	2.38 [0.81; 6.99]
Hengg 2019	-1.0688	1.1285	4.4%	0.34 [0.04; 3.14]
Kim 2022	-0.8522	1.1984	3.9%	0.43 [0.04; 4.47]
Lee 2019	-2.0541	1.4769	2.7%	0.13 [0.01; 2.32]
Liu 2021	-0.5108	1.3904	3.0%	0.60 [0.04; 9.16]
Ma 2024	-2.6671	1.4536	2.8%	0.07 [0.00; 1.20]
Opperman 2023	0.0000	1.3540	3.2%	1.00 [0.07; 14.21]
Pan 2021	-1.4959	1.6078	2.3%	0.22 [0.01; 5.23]
Sheng 2023	0.0000	0.9837	5.5%	1.00 [0.15; 6.88]
Siebenbürger 2019	-1.2694	1.5351	2.5%	0.28 [0.01; 5.69]
Wang 2019	-0.5281	0.8382	7.1%	0.59 [0.11; 3.05]
Wang 2023	-0.2865	0.5411	13.1%	0.75 [0.26; 2.17]
Zhang 2019	-1.7083	1.5331	2.5%	0.18 [0.01; 3.66]
Zhao 2019	-1.9459	1.4808	2.7%	0.14 [0.01; 2.60]
Zhu 2014	0.0953	3.2333	0.6%	1.10 [0.00; 621.76]
**Total (95% CI)**			**100.0%**	**0.49 [0.33; 0.74]**

Heterogeneity: $Tau^2 = 0.1980$; $Chi^2 = 16.23$, df = 20 ($P = 0.70$); $I^2 = 0\%$

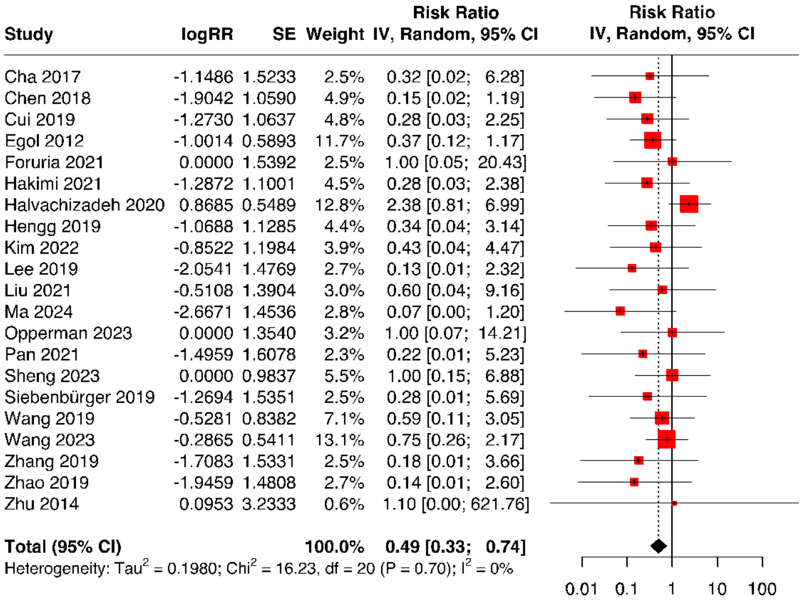

C. Risk of avascular necrosis of the humeral head

Study	logRR	SE	Weight	Risk Ratio IV, Random, 95% CI
Cha 2017	0.0000	2.8362	0.5%	1.00 [0.00; 259.50]
Chen 2018	0.3983	0.6987	8.9%	1.49 [0.38; 5.86]
Cui 2019	0.3365	0.9651	4.6%	1.40 [0.21; 9.28]
Davids 2020	0.0000	2.2331	0.9%	1.00 [0.01; 79.58]
Egol 2012	0.2513	1.2060	3.0%	1.29 [0.12; 13.67]
Foruria 2021	0.3254	0.5854	12.6%	1.38 [0.44; 4.36]
Hakimi 2021	0.0991	1.4001	2.2%	1.10 [0.07; 17.17]
Halvachizadeh 2020	-0.8543	1.0113	4.2%	0.43 [0.06; 3.09]
Hengg 2019	0.4353	0.8796	5.6%	1.55 [0.28; 8.66]
Katthagen 2018	1.0986	1.6078	1.7%	3.00 [0.13; 70.09]
Kim 2020	0.0000	2.2680	0.8%	1.00 [0.01; 85.21]
Kim 2022	-1.2552	1.6136	1.7%	0.29 [0.01; 6.74]
Lee 2019	1.2417	1.6204	1.6%	3.46 [0.14; 82.90]
Liu 2011	-0.3228	1.3849	2.3%	0.72 [0.05; 10.93]
Liu 2021	-0.5108	1.3904	2.2%	0.60 [0.04; 9.16]
Myers 2020	0.8589	0.8484	6.0%	2.36 [0.45; 12.45]
Opperman 2023	-0.6931	0.7638	7.4%	0.50 [0.11; 2.23]
Pan 2021	-0.4055	2.5658	0.7%	0.67 [0.00; 101.84]
Sheng 2023	0.0000	0.9837	4.5%	1.00 [0.15; 6.88]
Siebenbürger 2019	0.3438	0.7892	7.0%	1.41 [0.30; 6.62]
Tuerxun 2020	-1.3157	0.6559	10.1%	0.27 [0.07; 0.97]
Wang 2013	1.9459	1.4954	1.9%	7.00 [0.37; 131.22]
Wang 2019	0.1651	1.3974	2.2%	1.18 [0.08; 18.24]
Wang 2023	-0.6431	1.2041	3.0%	0.53 [0.05; 5.57]
Zhang 2019	-1.1975	1.6178	1.7%	0.30 [0.01; 7.19]
Zhao 2019	0.0000	1.3801	2.3%	1.00 [0.07; 14.95]
Zhu 2014	0.0000	3.1623	0.4%	1.00 [0.00; 491.74]
**Total (95% CI)**			**100.0%**	**0.98 [0.73; 1.32]**

Heterogeneity: $Tau^2 = 0$; $Chi^2 = 12.23$, df = 26 (P = 0.99); $I^2 = 0\%$

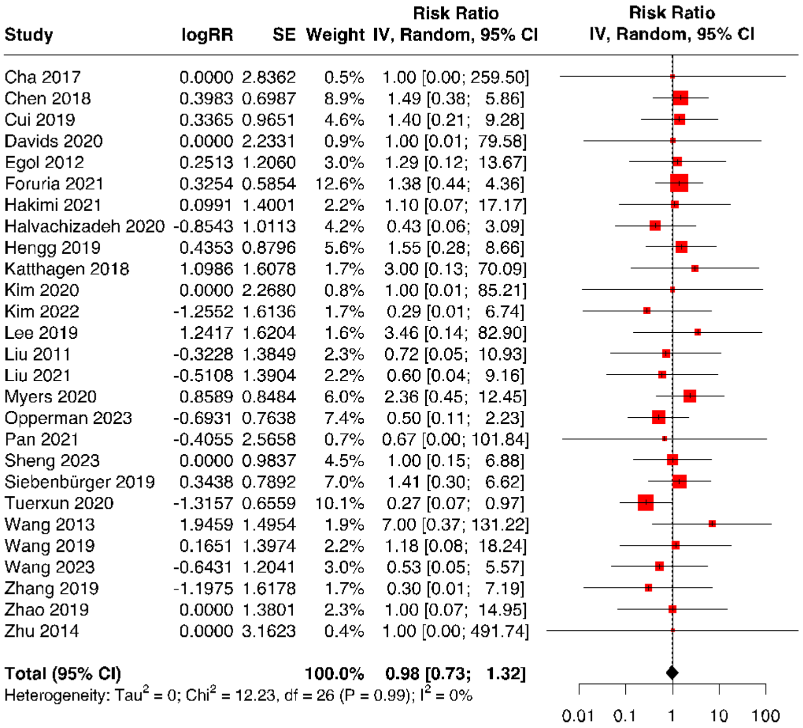

D. Pain

Study	SMD	SE	Weight	Std. Mean Difference IV, Random, 95% CI
Chen 2018	−0.6789	0.2183	4.2%	−0.68 [−1.11; −0.25]
Cui 2019	−0.6265	0.2680	3.7%	−0.63 [−1.15; −0.10]
Davids 2020	−0.5200	0.2274	4.1%	−0.52 [−0.97; −0.07]
Foruria 2021	−0.3684	0.1560	4.7%	−0.37 [−0.67; −0.06]
Hakimi 2021	0.1137	0.1994	4.3%	0.11 [−0.28; 0.50]
Halvachizadeh 2020	0.0320	0.2206	4.1%	0.03 [−0.40; 0.46]
Hengg 2019	0.0970	0.2445	3.9%	0.10 [−0.38; 0.58]
Katthagen 2018	−0.0065	0.2887	3.5%	−0.01 [−0.57; 0.56]
Kim 2020	0.0951	0.2281	4.1%	0.10 [−0.35; 0.54]
Kim 2022	−0.7666	0.2618	3.7%	−0.77 [−1.28; −0.25]
Lee 2019	−0.5396	0.2073	4.3%	−0.54 [−0.95; −0.13]
Liu 2011	−1.1005	0.3069	3.3%	−1.10 [−1.70; −0.50]
Liu 2021	−0.3853	0.2604	3.8%	−0.39 [−0.90; 0.13]
Liu 2023	−0.6376	0.2996	3.4%	−0.64 [−1.22; −0.05]
Ma 2024	0.0515	0.2034	4.3%	0.05 [−0.35; 0.45]
Opperman 2023	0.2404	0.4097	2.5%	0.24 [−0.56; 1.04]
Pan 2021	−1.2083	0.3129	3.3%	−1.21 [−1.82; −0.59]
Sheng 2023	0.0943	0.1797	4.5%	0.09 [−0.26; 0.45]
Siebenbürger 2019	−0.1369	0.2096	4.2%	−0.14 [−0.55; 0.27]
Tuerxun 2020	−0.5285	0.2684	3.7%	−0.53 [−1.05; −0.00]
Wang 2013	−0.7466	0.2313	4.0%	−0.75 [−1.20; −0.29]
Wang 2019	−1.2158	0.2368	4.0%	−1.22 [−1.68; −0.75]
Wang 2023	−0.0640	0.2237	4.1%	−0.06 [−0.50; 0.37]
Zhang 2019	−0.7053	0.2307	4.0%	−0.71 [−1.16; −0.25]
Zhao 2019	−0.1903	0.3093	3.3%	−0.19 [−0.80; 0.42]
Zhu 2014	−1.5619	0.3626	2.9%	−1.56 [−2.27; −0.85]
**Total (95% CI)**			**100.0%**	**−0.41 [−0.60; −0.23]**

Heterogeneity: $Tau^2 = 0.1433$; $Chi^2 = 83.86$, df = 25 (P < 0.01); $I^2 = 70\%$

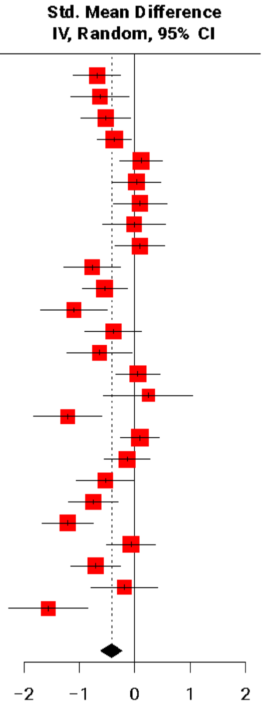

E. Function

Study	SMD	SE	Weight	Std. Mean Difference IV, Random, 95% CI
Chen 2018	0.6789	0.2183	4.2%	0.68 [0.25; 1.11]
Cui 2019	0.6265	0.2680	3.7%	0.63 [0.10; 1.15]
Davids 2020	0.5200	0.2274	4.1%	0.52 [0.07; 0.97]
Foruria 2021	0.3684	0.1560	4.7%	0.37 [0.06; 0.67]
Hakimi 2021	−0.1137	0.1994	4.3%	−0.11 [−0.50; 0.28]
Halvachizadeh 2020	0.2026	0.2209	4.1%	0.20 [−0.23; 0.64]
Hengg 2019	−0.0970	0.2445	3.9%	−0.10 [−0.58; 0.38]
Katthagen 2018	−0.0065	0.2887	3.5%	−0.01 [−0.57; 0.56]
Kim 2020	−0.4192	0.2304	4.0%	−0.42 [−0.87; 0.03]
Kim 2022	0.7666	0.2618	3.8%	0.77 [0.25; 1.28]
Lee 2019	0.7050	0.2098	4.2%	0.70 [0.29; 1.12]
Liu 2011	1.1005	0.3069	3.4%	1.10 [0.50; 1.70]
Liu 2021	0.6038	0.2637	3.8%	0.60 [0.09; 1.12]
Liu 2023	0.6376	0.2996	3.4%	0.64 [0.05; 1.22]
Ma 2024	0.7047	0.2095	4.2%	0.70 [0.29; 1.12]
Opperman 2023	0.5852	0.4169	2.6%	0.59 [−0.23; 1.40]
Pan 2021	1.7131	0.3357	3.1%	1.71 [1.06; 2.37]
Sheng 2023	0.3375	0.1809	4.5%	0.34 [−0.02; 0.69]
Siebenbürger 2019	0.1369	0.2096	4.2%	0.14 [−0.27; 0.55]
Tuerxun 2020	0.5285	0.2684	3.7%	0.53 [0.00; 1.05]
Wang 2013	0.7466	0.2313	4.0%	0.75 [0.29; 1.20]
Wang 2019	1.2158	0.2368	4.0%	1.22 [0.75; 1.68]
Wang 2023	−0.0197	0.2237	4.1%	−0.02 [−0.46; 0.42]
Zhang 2019	0.4500	0.2267	4.1%	0.45 [0.01; 0.89]
Zhao 2019	0.7515	0.3193	3.3%	0.75 [0.13; 1.38]
Zhu 2014	1.5619	0.3626	2.9%	1.56 [0.85; 2.27]
**Total (95% CI)**			**100.0%**	**0.52 [0.33; 0.71]**

Heterogeneity: $Tau^2 = 0.1558$; $Chi^2 = 86.21$, df = 25 ($P < 0.01$); $I^2 = 71\%$

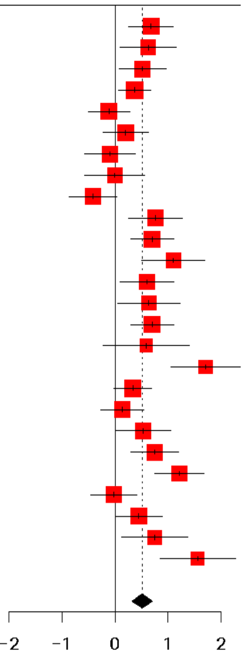

F. Change of humeral head height

Study	MD	SE	Weight	Mean Difference IV, Random, 95% CI
Cha 2017	−3.0000	0.7416	6.9%	−3.00 [−4.45; −1.55]
Cui 2019	−2.9800	0.7421	6.9%	−2.98 [−4.43; −1.53]
Lee 2019	−2.4000	0.0512	10.8%	−2.40 [−2.50; −2.30]
Liu 2021	−2.0600	0.3734	9.5%	−2.06 [−2.79; −1.33]
Ma 2024	−1.5000	0.0979	10.7%	−1.50 [−1.69; −1.31]
Pan 2021	−0.3600	0.1392	10.6%	−0.36 [−0.63; −0.09]
Peng 2012	−3.9000	0.2321	10.3%	−3.90 [−4.35; −3.45]
Sheng 2023	−1.2400	0.4392	9.0%	−1.24 [−2.10; −0.38]
Tuerxun 2020	−2.3000	0.4597	8.9%	−2.30 [−3.20; −1.40]
Wang 2023	−0.7000	0.9266	5.7%	−0.70 [−2.52; 1.12]
Zhao 2019	−2.4000	0.1166	10.7%	−2.40 [−2.63; −2.17]
**Total (95% CI)**			**100.0%**	**−2.08 [−2.78; −1.37]**

Heterogeneity: $Tau^2 = 0.9656$; $Chi^2 = 303.81$, df = 10 ($P < 0.01$); $I^2 = 97\%$

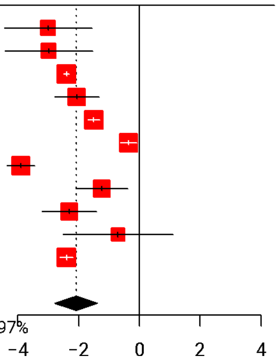

G. Change of neck-shaft angle

Study	MD	SE	Weight	Mean Difference IV, Random, 95% CI
Cha 2017	-7.2000	1.4076	6.3%	-7.20 [-9.96; -4.44]
Chen 2018	-8.4000	3.4983	2.9%	-8.40 [-15.26; -1.54]
Cui 2019	-6.8000	1.7846	5.5%	-6.80 [-10.30; -3.30]
Kim 2020	-2.5000	1.7536	5.6%	-2.50 [-5.94; 0.94]
Kim 2022	-11.2000	2.4131	4.4%	-11.20 [-15.93; -6.47]
Lee 2019	-7.1000	0.1768	8.0%	-7.10 [-7.45; -6.75]
Liu 2021	-2.3900	0.8644	7.2%	-2.39 [-4.08; -0.70]
Liu 2023	-2.6000	1.0749	6.9%	-2.60 [-4.71; -0.49]
Ma 2024	-5.9000	0.3343	7.9%	-5.90 [-6.56; -5.24]
Myers 2020	-2.9000	1.2319	6.6%	-2.90 [-5.31; -0.49]
Pan 2021	-10.8000	2.0797	5.0%	-10.80 [-14.88; -6.72]
Peng 2012	-8.7000	0.6039	7.6%	-8.70 [-9.88; -7.52]
Sheng 2023	-2.7500	0.8970	7.2%	-2.75 [-4.51; -0.99]
Tuerxun 2020	-4.3000	0.9591	7.1%	-4.30 [-6.18; -2.42]
Wang 2023	2.3000	2.6341	4.0%	2.30 [-2.86; 7.46]
Zhao 2019	-6.4000	0.2664	7.9%	-6.40 [-6.92; -5.88]
**Total (95% CI)**			**100.0%**	**-5.39 [-7.10; -3.67]**

Heterogeneity: $Tau^2 = 7.0819$; $Chi^2 = 120.20$, df = 15 ($P < 0.01$); $I^2 = 88\%$

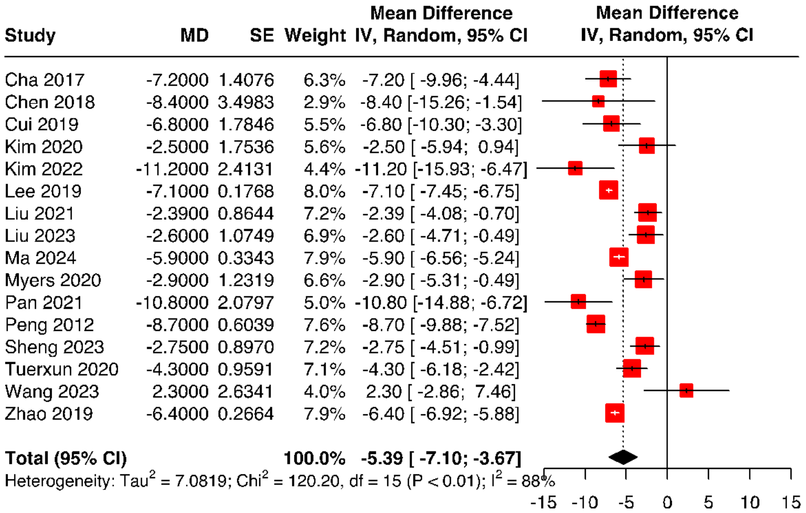

The size of the red box was determined by the weight of the study in the random-effects meta-analysis.
Abbreviations: CI, confidence interval; IV, inverse variance; RR, relative risk; SE, standard error.

Figure S2. Contour-enhanced funnel plots

A. Pain

Before the trim and fill method

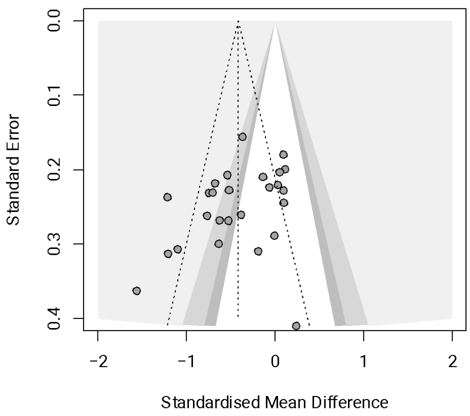

After the trim and fill method

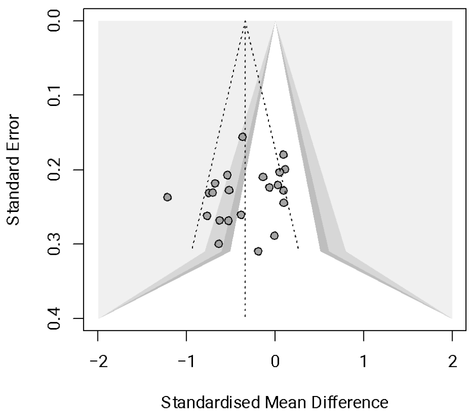

B. Function

Before the trim and fill method

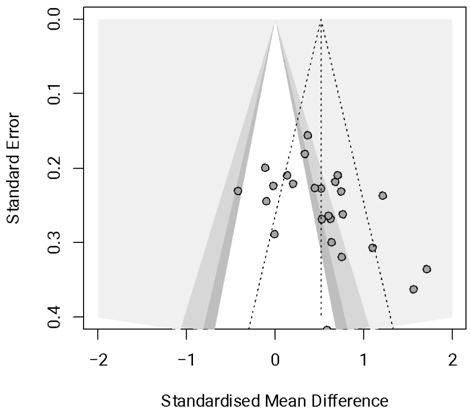

After the trim and fill method

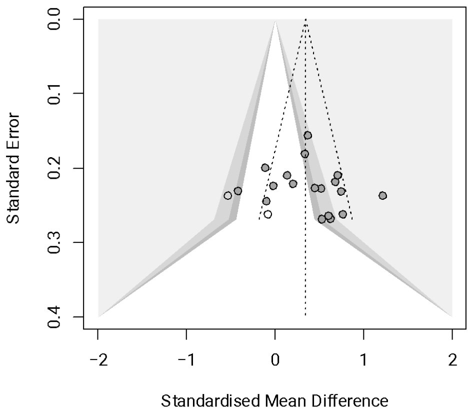

C. Change of humeral head height

Before the trim and fill method

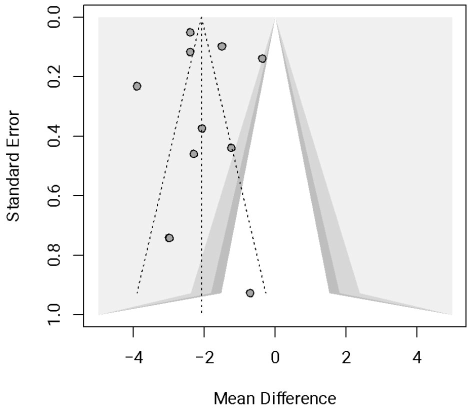

No significant small study bias

D. Change of neck-shaft angle

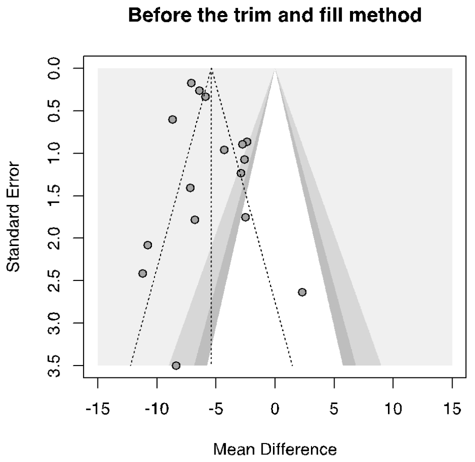

No significant small study bias

The contour from dark gray to light gray respectively showed the regions of p <0.1, <0.05, and <0.01. Each black dot represents an effect estimate of a study. The dashed line indicates the effect estimate of the meta-analysis. The white dot represents a hypothetical study added by the trim and fill method.
